# *Combi*-CRISPR: combination of NHEJ and HDR provides efficient and precise plasmid-based knock-ins in mice and rats

**DOI:** 10.1007/s00439-020-02198-4

**Published:** 2020-07-02

**Authors:** Kazuto Yoshimi, Yuichiro Oka, Yoshiki Miyasaka, Yuko Kotani, Misato Yasumura, Yoshihiro Uno, Kosuke Hattori, Arisa Tanigawa, Makoto Sato, Manami Oya, Kazuhiro Nakamura, Natsuki Matsushita, Kazuto Kobayashi, Tomoji Mashimo

**Affiliations:** 1 Division of Animal Genetics, Laboratory Animal Research Center, Institute of Medical Science, The University of Tokyo, Tokyo, 108-8639 Japan; 2 Division of Genome Engineering, Center for Experimental Medicine and Systems Biology, Institute of Medical Science, The University of Tokyo, Tokyo, 108-8639 Japan; 3 Department of Anatomy and Neurosciences, Graduate School of Medicine, Osaka University, Osaka, 565-0871 Japan; 4 Department of Child Development, United Graduate School of Child Development, Osaka University, Osaka, 565-0871 Japan; 5 Institute of Experimental Animal Sciences, Graduate School of Medicine, Osaka University, Osaka, 565-0871 Japan; 6 Department of Integrative Physiology, Nagoya University Graduate School of Medicine, Nagoya, 466-8550 Japan; 7 Division of Laboratory Animal Research, Aichi Medical University School of Medicine, Aichi, 480-1195 Japan; 8 Department of Molecular Genetics, Institute of Biomedical Sciences, Fukushima Medical University School of Medicine, Fukushima, 960-1295 Japan; 9 Genome Editing Research and Development Center, Graduate School of Medicine, Osaka University, Osaka, 565-0871 Japan

## Abstract

CRISPR-Cas9 are widely used for gene targeting in mice and rats. The non-homologous end-joining (NHEJ) repair pathway, which is dominant in zygotes, efficiently induces insertion or deletion (indel) mutations as gene knockouts at targeted sites, whereas gene knock-ins (KIs) via homology-directed repair (HDR) are difficult to generate. In this study, we used a double-stranded DNA (dsDNA) donor template with Cas9 and two single guide RNAs, one designed to cut the targeted genome sequences and the other to cut both the flanked genomic region and one homology arm of the dsDNA plasmid, which resulted in 20–33% KI efficiency among G0 pups. G0 KI mice carried NHEJ-dependent indel mutations at one targeting site that was designed at the intron region, and HDR-dependent precise KIs of the various donor cassettes spanning from 1 to 5 kbp, such as *EGFP*,* mCherry*,* Cre*, and genes of interest, at the other exon site. These findings indicate that this combinatorial method of NHEJ and HDR mediated by the CRISPR-Cas9 system facilitates the efficient and precise KIs of plasmid DNA cassettes in mice and rats.

**Electronic supplementary material:**

The online version of this article (10.1007/s00439-020-02198-4) contains supplementary material, which is available to authorized users.

## Background

Zinc finger nucleases (ZFNs), transcription activator-like effector nucleases (TALENs), and clustered regularly interspaced short palindromic repeats (CRISPR)/CRISPR associated protein 9 (Cas9) enable rapid and precise genetic manipulation in mammalian cells (Gaj et al. 2013; Peng et al. 2014). Recently, the introduction of the CRISPR-Cas9 system has enabled the knockout of genes in zygotes via non-homologous end-joining (NHEJ) with unprecedented simplicity and speed (Mashimo 2014; Peng et al. 2014; Sander and Joung 2014; Wang et al. 2013). Multiple gene knockouts can also be achieved using several different sgRNAs designed to target multiple genes (Wang et al. 2013; Yoshimi et al. 2014). More recently, several groups, including ours, reported genome engineering using the zygote electroporation of CRISPR-Cas9, which represents an easy and rapid alternative to the elaborate pronuclear injection procedure for genome editing in mice and rats (Kaneko et al. 2014; Qin et al. 2015).

CRISPR-Cas9-mediated knock-ins (KIs) in zygotes have been achieved via homology-directed repair (HDR) with a donor DNA template (Yang et al. 2013). Either microinjection or electroporation of CRISPR-Cas9 together with a single-stranded oligodeoxynucleotide (ssODN) has become a widely used method to introduce point mutations or short tag sequences in zygotes (Inui et al. 2014; Yang et al. 2013). The use of long ssDNA (lssDNA) has been developed as an efficient alternative donor template for the CRISPR-Cas9-mediated KIs of cassette sequences or two loxP sites (Miura et al. 2015; Yoshimi et al. 2016). *Quadros *et al*.* reported Easi-CRISPR for creating KI or conditional knockout mouse models using lssDNA produced by in vitro transcription and reverse transcription or obtained from the company (Quadros et al. 2017). We also reported a CLICK method using lssDNA purified from nicked dsDNA plasmids for the manipulation of GFP cassette sequences (Yoshimi et al. 2016), or for the quick generation of conditional knockout mice (Miyasaka et al. 2018). However, these approaches provide less efficiency or incomplete KIs when more than 2 kb sequences of lssDNA are used as a donor template.

Using double-stranded DNA (dsDNA) as a donor template, the HDR-mediated KI efficiency is usually very low in zygotes. A cloning-free method, the direct nuclear delivery of Cas9 protein complex with chemically synthesized dual RNAs, was investigated for the efficient generation of knock-in mice (Aida et al. 2015). Several approaches to improve the HDR efficiency include chemical reagents (Song et al. 2016) or small molecules (Maruyama et al. 2015). Other approaches have used the stabilization of ssODNs (Renaud et al. 2016) and sgRNAs (Hendel et al. 2015) by chemical modification. Several other technical approaches, including homology-independent targeted integration (HITI) (Suzuki et al. 2016), obligate ligation-gated recombination (ObLiGaRe) (Auer et al. 2014; Maresca et al. 2013), and precise integration into target chromosome (PITCH) (Nakade et al. 2014) have been reported, some of which were efficient in cultured or in vivo cells. Recently, homology-mediated end-joining (HMEJ) (Yao et al. 2017) and targeted integration with linearized dsDNA (Tild)-CRISPR (Yao et al. 2018) were shown to provide efficient gene KIs in mouse and human cells, but some of these methods were not efficient in zygotes, or have not been well evaluated. Recently, two-cell homologous recombination (2C-HR), a method based on introducing CRISPR reagents into embryos at the two-cell stage, has been reported as an efficient gene-integration approach in zygotes (Gu et al. 2018).

In this study, we report a new powerful method of generating plasmid-based KI in mice and rats using the CRISPR-Cas9 system. The principle concept of this method is the combination of highly efficient editing via NHEJ and the low efficiency, but precise editing of HDR in zygotes. We termed this approach *Combi*-CRISPR, and the combination of NHEJ and HDR provides efficient and precise knock-ins of large DNA fragments in mice and rats. A similar concept to our approach was recently reported whereby the combination of NHEJ and HDR (termed SATI) efficiently integrated transgenes in a targeted manner (Suzuki et al. 2019). Our approach has improved the SATI method further by delivering a second sgRNA to enhance HDR.

## Results

### Generation of plasmid-based knock-in mice with CRISPR-Cas9 and two sgRNAs

**Fig. 1:**
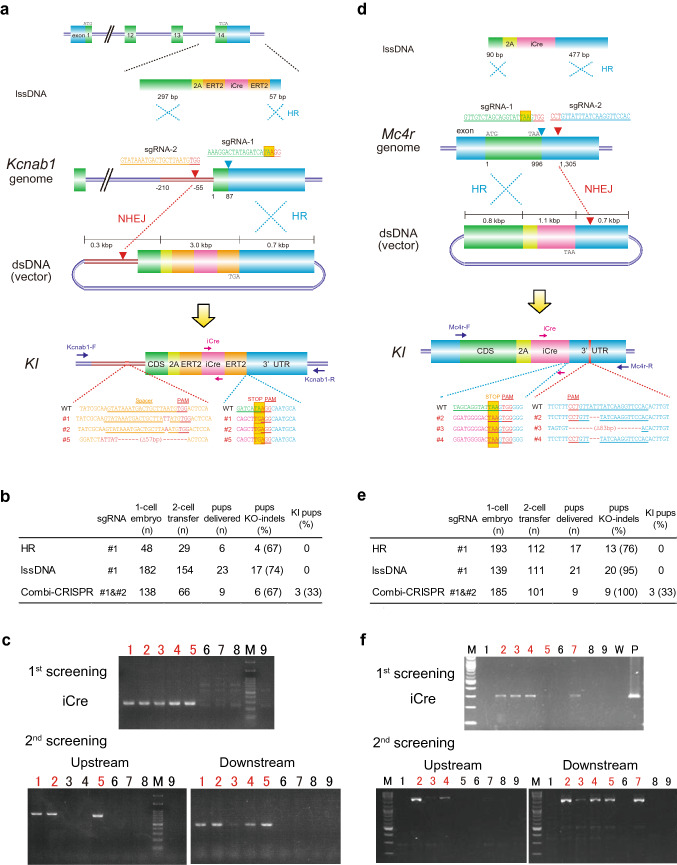
Injection of two sgRNAs, Cas9, and a donor dsDNA into mouse zygotes. **a** Methods to integrate the P2A-ERT2-iCre-ERT2 cassette at the terminal codon of the *Kcnab1* gene with lssDNA (above) or dsDNA (bottom). Microinjection of two sgRNAs, Cas9, and dsDNA provided three KI mice (#1, 2, and 5) carrying precise KIs of the iCre cassette at the sgRNA-1 targeting site and insertion or deletion mutations at the sgRNA-2 targeting site. **b**, **e** Comparison of three methods using dsDNA with single sgRNA-1 (HR), lssDNA with sgRNA-1 (lssDNA), or dsDNA with two sgRNAs (*Combi*-CRISPR) for KIs in mouse zygotes. **c**, **f** PCR analysis using primer sets amplifying the internal region of the iCre cassette (first screening) or for 5′ genome-donor boundary (Upstream) and donor-3′ genome boundary (Downstream in second screening) in delivered mouse pups (#1–9 for **c** and #1–5 for **f**). M: 100 bp DNA ladder marker. **d** Methods to integrate P2A-iCre cassette at the terminal codon of the *Mc4r* gene with lssDNA (above) or dsDNA (bottom). Microinjection of two sgRNA, Cas9, and dsDNA provided three KI mice (#2–4) carrying precise KIs of the iCre cassette at the sgRNA-1 targeting site and several deletion mutations at the sgRNA-2 targeting site

sgRNA-1 was designed at the terminal codon of the potassium voltage-gated channel subfamily A member regulatory beta subunit 1 (*Kcnab1*) gene to integrate a bi-cistronic expression cassette encoding tamoxifen-inducible Cre-recombinase (Fig. 1a and Supplementary Table 1). We prepared a dsDNA donor vector including a 2.9 kbp P2A-ERT2-iCre-ERT2 cassette with a 297 bp 5′ homology arm (HA) and a 695 bp 3′ HA, or lssDNA donor with a 297 bp 5′ HA and a 57 bp 3′ HA, which was purified according to our previously reported method (Yoshimi et al. 2016) (Fig. 1a). We injected dsDNA (3 ng/µl) or lssDNA (40 ng/µl) with Cas9 mRNA (20 ng/µl) and sgRNA-1 (25 ng/µl) into mouse C57BL/6 embryos, which resulted in 4 of 6 delivered pups and 17 of 23 delivered pups, respectively, carrying indel mutations at the sgRNA-1 target site (Fig. 1b). However, no KI mouse with dsDNA or lssDNA was obtained via conventional HDR.

**Table 1:** Generation of knock-in mice with CRISPR-Cas9

Targeting	Method	Vector size (bp)	Eggs	Two-cell	Pups	KO	KI
*Ctgf-ERT2-iCre-ERT2*	lssDNA	3345	177	151	18	13	0
	HR	4563	50	38	7	ND	0
	Combi-CRISPR	4563	151	59	6	ND	1
*Slc12a1-iCre*	lssDNA	1493	997	898	220	42	1
	Combi-CRISPR	2662	197	68	6	4	1
*Bmi1-mCherry*	lssDNA	1685	340	327	71	38	0
	Combi-CRISPR	2984	165	117	22	14	2
*Plxnd1-ERT2-iCre-ERT2*	Combi-CRISPR	5232	165	114	9	6	2
*Cdkn2a-tdT-DTR*	Combi-CRISPR	4136	246	130	17	10	1
*Cdkn2a-CD2-DTR*	Combi-CRISPR	3459	239	136	10	6	2

To increase the knock-in efficiency, we designed an additional sgRNA-2 within intron 13 (55 bp upstream of exon 14) to cut the second site of the genomic region and the homologous region of the donor dsDNA plasmid (Fig. 1a and Supplementary Table 1). Injection of Cas9 mRNA (50 ng/µl), two sgRNAs (sgRNA-1 and sgRNA-2, 25 ng/µl each), and the donor dsDNA (1 ng/µl) into C57BL/6 embryos resulted in nine delivered pups. First screening by PCR and sequencing with primers amplifying the sgRNA-1 target region and the internal region of the iCre cassette (Supplementary Table 2) revealed six pups carrying indel mutations (KO) and five pups carrying a P2A-ERT2-iCre-ERT2 insertion (Fig. 1c). Second PCR and sequencing analysis using primer sets for the 5′ genome-donor boundary and donor-3′ genome boundary identified three KI mice that carried indel mutations, such as a 2-bp insertion or 57 bp deletion at the intron region (Upstream) and precise KI of the P2A-ERT2-iCre-ERT2 cassette at the terminal codon of the *Kcnab1* gene (Downstream; Fig. 1a–c and Supplementary Table 2).

Crossing the KI founder mouse with a wild-type C57BL/6 mouse confirmed the germline transmission of the KI allele in F1 KI mice (Supplementary Fig. 1a). To further evaluate whether the ERT2-iCre-ERT2 allele was functional, we crossed the founder KI mice with reporter mice (B6.Cg-Gt(ROSA)26Sor^tm14(CAG-tdTomato)Hze^/J: Jackson 007914), which provided inducible recombination events at the flox site and tdTomato expression in the neurons of F1 KI mice (Supplementary Fig. 1b, c).

### Combination of the NHEJ and HDR pathways to induce efficient knock-ins

To duplicate this KI approach, we targeted the melanocortin 4 receptor (*Mc4r*) gene with the T2A-iCre bi-cistronic expressing vector. We designed sgRNA-1 targeting the terminal codon of the *Mc4r* gene and sgRNA-2 cutting both the 3′ untranslated region (UTR) and the 696 bp 3′ HA of the donor plasmid (Fig. 1d and Supplementary Table 1). Injection of sgRNA-1, sgRNA-2 (25 ng/µl each), Cas9 mRNA (20 ng/µl), and the donor vector (2 ng/µl) into C57BL/6 mouse embryos resulted in nine pups, five of which carried the iCre cassette at the first screening (Fig. 1e, f). Second PCR and sequencing with the boundary primer sets (Supplementary Table 2) revealed three mice carrying indel mutations at the sgRNA-2 targeting site and a precise KI allele of the P2A-iCre cassette at the sgRNA-2 targeting site (Fig. 1d–f). We also repeated the other approaches using a conventional dsDNA targeting vector or lssDNA donor template with sgRNA-1/Cas9. However, no KI mouse was obtained among similar numbers of delivered pups by these two methods (Fig. 1e).

To evaluate the efficiency of this method using two sgRNAs/dsDNA compared with the other methods, we targeted five other genomic loci and six different donor vectors from 2.6 to 5.2 kbp in size including *Cre*, inducible *Cre*, *mCherry*, and diphtheria toxin receptor (*DTR*) genes (Table 1). Overall, nine KI mice were obtained among 70 delivered pups, indicating 13% efficiency, although no KI mouse was identified when using the lssDNA methods. In the KI mice generated by the two sgRNAs method, we often observed various indel mutations at the sgRNA-2 targeting sites that were designed in the intron region. We also found precise KI alleles at the sgRNA-1 targeting sites that were designed at the terminal codon of each gene. Therefore, from the sequence data of the KI founder mice, we speculated that indel mutations at the sgRNA-2 targeting sites were repaired via NHEJ and KI alleles were precisely repaired via HDR between the sgRNA-1 targeting genome and the donor plasmid (Fig. 3). We termed this KI method using two sgRNAs/Cas9 and a dsDNA donor *Combi*-CRISPR (combination of NHEJ and HR repair pathway to induce efficient knock-ins).

### Generation of KI rats using the *Combi*-CRISPR method

**Fig. 2:**
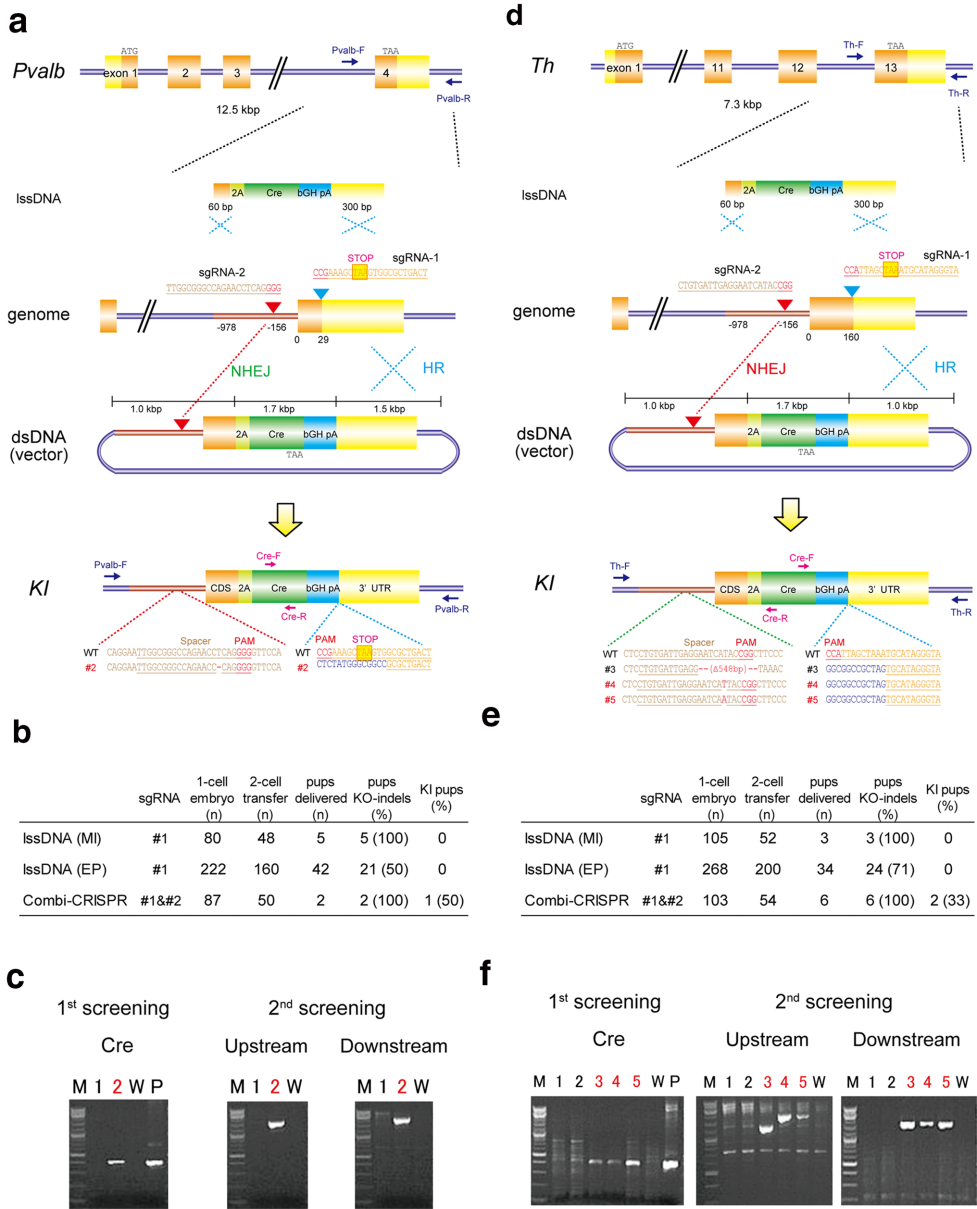
Knock-in rats generated by injection of two sgRNAs, Cas9, and a donor dsDNA in rat zygotes. **a** Methods to integrate the P2A-Cre cassette at the terminal codon of the *Pvalb* gene with lssDNA (above) or dsDNA (bottom). Microinjection of two sgRNA, Cas9, and dsDNA provided a KI rat (#1) carrying precise KIs of the Cre cassette at the sgRNA-1 targeting site and a 1 bp deletion mutation at the sgRNA-2 targeting site. **b**, **e** Comparison of three methods using dsDNA with single sgRNA-1 (HR), lssDNA with sgRNA-1 (lssDNA), or dsDNA with two sgRNAs (*Combi*-CRISPR) for KIs in rat zygotes. **c**,** f** PCR analysis using primer sets amplifying the internal region of the Cre cassette (first screening) or for 5′ genome-donor boundary (Upstream) and donor-3′ genome boundary (Downstream in second screening) in delivered rat pups (#1–2 for **c** and #1–5 for **f**). M: 100 bp DNA ladder marker. **d** Methods to integrate the P2A-Cre cassette at the terminal codon of the *Th* gene with lssDNA (above) or dsDNA (bottom). Microinjection of two sgRNA, Cas9, and dsDNA provided two KI rats (#4, 5) carrying precise KIs of the Cre cassette at the sgRNA-1 targeting site and insertion or deletion mutations at the sgRNA-2 targeting site. Integration of the P2A-Cre cassette with the 3′ HA, and confirmation of the vector sequences by PCR and sequencing analysis in #3 rat

To examine whether the *Combi*-CRISPR method could be applied to rat zygotes, we targeted the terminal exon of the parvalbumin (*Pvalb*) gene and tyrosine hydroxylase (*Th*) gene to integrate a 1.7 kbp P2A-Cre cassette for the bi-cistronic expression of Cre-recombinase in rats. We prepared dsDNA donor vectors with 1–1.5 kbps HAs or lssDNA with 60–300 bps HAs for the two genes (Fig. 2a, d). We also prepared sgRNA-1 targeting the terminal codon of the *Pvalb* or *Th* gene and sgRNA-2 targeting the intron upstream of the terminal exon. First, we examined the lssDNA method for the two genes. Microinjection or electroporation of lssDNA and Cas9/sgRNA-1 into F344 rat embryos resulted in 47 pups with the *Pvalb* gene and 37 pups with the *Th* gene (Fig. 2b, e). Overall, 26 KO pups for the *Pvalb* gene and 27 pups for the *Th* gene were obtained, but no KI rat was identified by PCR analysis for the two genes. However, the microinjection of dsDNA, Cas9, and two sgRNA-1 and sgRNA-2, into 197 F344 rat embryos for the *Pvalb* gene and 182 embryos for the *Th* gene resulted in two and six pups delivered, respectively. Of these, one (#2) and three (#3, #4 and #5) KIs were obtained, respectively, by the first PCR screening with primers amplifying the sgRNA-1 target region and the internal region of the *Cre* cassette (Fig. 2b, e).

**Fig. 3:**
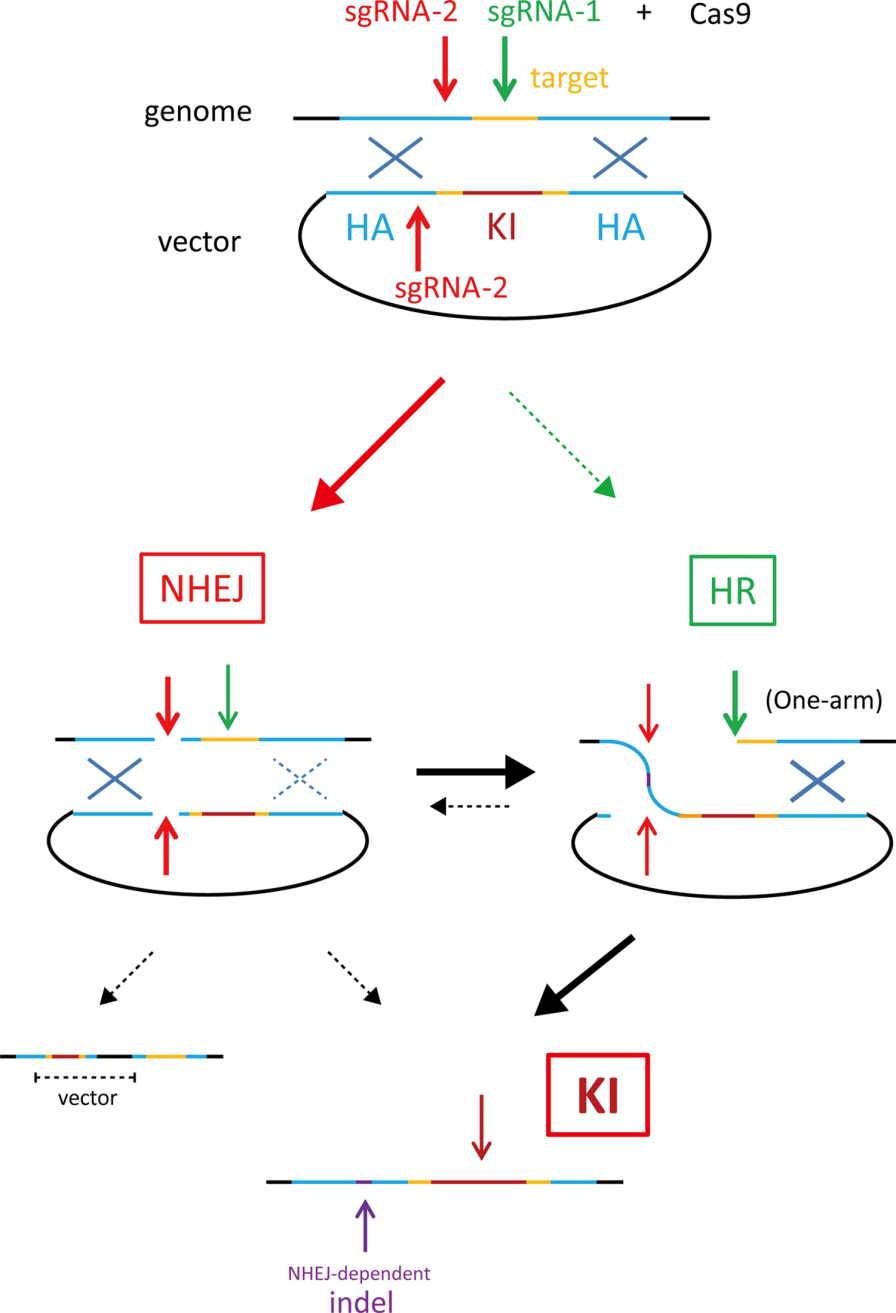
Schematic representation of precise and efficient knock-ins by *Combi*-CRISPR. A dsDNA donor vector was used with Cas9 and two sgRNAs, one designed to cut the targeted genome sequences (sgRNA-2) and the other to cut both the flanked genomic region and one homology arm of the dsDNA plasmid (sgRNA-1 targeting). The NHEJ repair pathway dominantly induces indel mutations (purple) at the sgRNA-2 targeting site. Thereafter, the HDR pathway integrates a KI cassette (red) without any mutation at the sgRNA-1 targeting site. In some cases, the whole donor vector was integrated at the sgRNA-2 targeting site via NHEJ (black)

Second PCR and sequencing analysis using primer sets amplifying the 5′ genome-donor boundary and donor 3′ genome boundary (Supplementary Table 2) indicated indel mutations at the sgRNA-2 targeting intron region and KI of the P2A-Cre cassette before the terminal codon of *Pvalb* (#2) and *Th* genes (#4 and #5). However, #3 rat carried the P2A-Cre cassette followed by the 3′ HA and the vector sequences, which indicates that the whole donor vector sequence was integrated via the NHEJ repair pathway alone (Fig. 3).

## Discussion

In this study, we report a novel CRISPR-mediated genome engineering method, *Combi*-CRISPR, which combines the NHEJ and HDR repair pathways for the efficient and precise KI of a few kbp gene cassette in mice and rats. It is necessary to prepare a donor dsDNA with hundreds to 1 kbps HAs and to design the first sgRNA (sgRNA-1) at the target site where the KI cassette should be integrated in the same way as for the conventional HDR-dependent KI method. It is also necessary to design another sgRNA (sgRNA-2) targeting an arbitrary region close to the first targeting site and within the HA region where the incidence of CRISPR-mediated mutations is minimized, although indels in intronic or 3′UTR regions might have serious consequences. Similarly, the main limit of our technique is that it is only applicable to a subset of KI strategies such as Tagging and cannot be used for other strategies, such as humanizing an animal gene, which cannot accommodate indels. Using this *Combi*-CRISPR method, we demonstrated efficient KIs using various types of donor cassettes, such as *EGFP*,* mCherry*,* Cre*, and genes of interest, for seven targeting loci in mice and two in rats. A similar approach was recently reported as HMEJ, which induced efficient recombination between two DSBs in the genomic region and the homology arms of the dsDNA donor, although the HMEJ-mediated repair mechanism remains unknown (Yao et al. 2017). Our PCR and sequencing analysis on the KI founders always indicated that indel mutations via NHEJ repair were present at the sgRNA-2 target site. In contrast, the precise KI of the donor cassettes without any small indel mutations via HDR were detected at the sgRNA-1 target site in all KI animals, except for one rat carrying a whole donor vector sequence, probably because plasmid integration occurred via NHEJ alone (Fig. 3). Therefore, we speculate that the first sgRNA-2/Cas9 induces double-strand breaks (DSBs), and then, indel mutations are induced via dominant NHEJ in zygotes at the first step. This first step may induce the assembly of several factors associated with the DSB repair pathway, which may then induce the efficient repair of the other DSB via HDR between the genomic target region and the homologous arm of the plasmid donor vector (Fig. 3).

There are several KI methods using the CRISPR-Cas9 system in mice and rats (Table 2). To exchange a single point mutation or introduce small indels, ssODNs are widely used as a donor template with CRISPR-Cas9. When sequences longer than 50 bp are to be integrated, the lssDNA can also be used in zygotes. Efficient KIs of simple *Cre* cassette sequences or flanked two loxP sites were previously reported by Easi-CRISPR in mice (Quadros et al. 2017) and by CLICK in rats (Miyasaka et al. 2018). The advantage of using ssDNA as a donor template is that electroporation can be used. However, both short and long ssDNA methods have size limitations for KIs, less than 100 bp and 2 kbp in zygotes, respectively. For KIs of longer cassette sequences, a conventional CRISPR-mediated KI method via HDR using a dsDNA donor template is available, although its efficiency is low. Several other KI methods using dsDNAs as donor templates were reported for cultured cells and mice (Auer et al. 2014; Gu et al. 2018; He et al. 2016; Maresca et al. 2013; Nakade et al. 2014; Suzuki et al. 2016, 2019) (Table 2). ObLiGaRe, HITI, PITCH, HMEJ, and SATI are useful for KI in in vitro cultures; however, these technologies have not been examined thoroughly in mouse and rat zygotes. Tild-CRISPR, based on an HMEJ strategy, was recently reported using linear dsDNA as a donor template (Yao et al. 2018). The *Combi*-CRISPR method uses circular dsDNA, although both technologies use sgRNA to cut the targeted genome sequences and the homology arm of the dsDNA, which might increase the recombination efficiency via HMEJ (Yao et al. 2018). 2C-HR is a unique, highly efficient, and useful technology for gene KI in zygotes, except for the technical difficulties for general researchers and technicians (Gu et al. 2018). Our *Combi*-CRISPR method provides an efficient and precise KI strategy in mouse and rat zygotes, which is suitable for projects that can accommodate indels in intronic or otherwise dispensable regions.

**Table 2:** Various knock-in methods in mouse zygotes with genome editing technology

Method		Repair	Donor	Vector cut	Homology arms	Efficiency	Accuracy	Size (bp)	References
Conventional HR		Homologous recombination (HR)	dsDNA	−	200–1 K	Low	High	1–10 K	Lin et al. (2018), Ma et al. (2014), Yang et al. (2013)
ssODN		Homology directed repair (HDR)	ssDNA	−	40–80	High	High	1–100	Wang et al. (2013), Yoshimi et al. (2014)
ObLiGaRe	Obligate ligation-gated recombination	Non-homologous end joining (NHEJ)	dsDNA	+	–	–	Low	1–10 K	Auer et al. (2014), He et al. (2016), Maresca et al. (2013)
PITCh	Precise integration into target chromosome	Microhomology-mediated end-joining (MMEJ)	dsDNA	+	20	Low	High	1–10 K	Nakade et al. (2014)
HITI	Homology-independent targeted integration	NHEJ	dsDNA	+	20	Low	Low	1–10 K	Suzuki et al. (2016)
SATI	intercellular linearized single homology arm donor-mediated intron-targeting integration	NHEJ and HR	dsDNA	+	200–1 K	High	High	1–10 K	Suzuki et al. (2019)
2H2OP	Two-hit and two oligos with a targeting plasmid	NHEJ?	dsDNA	+	–	Low	Low	1–200 K	Yoshimi et al. (2016)
2C-HR	Two-cell homologous recombination	HDR	dsDNA	−	200–1 K	High	High	1–10 K	Gu et al. (2018)
HMEJ	Homology-mediated end joining	NHEJ? or HR?	dsDNA	+	200–1 K	Low	Low	1–10 K	Yao et al. (2017), Zhang et al. (2017)
Tild-CRISPR	Targeted integration with linearized dsDNA-CRISPR	NHEJ? or HR?	dsDNA	linear	200–1 K	High	Low	1–10 K	Yao et al. (2018)
Easi-CRISPR	Efficient additions with ssDNA inserts-CRISPR	HDR	long ssDNA	−	50–300	High	High	1–2 K	Quadros et al. (2017)
CLICK	CRISPR with lssDNA inducing conditional knockout alleles	HDR	long ssDNA	−	50–300	High	High	1–2 K	Miyasaka et al. (2018)
Combi-CRISPR	Combination of NHEJ and HR with CRISPR	NHEJ and HR	dsDNA	+	200–1 K	High	High	1–10 K	This manuscript

In this study, *Combi*-CRISPR provided efficient KIs of approximately 10–33% in zygotes. However, there are some disadvantages compared with other KI methods. *Combi*-CRISPR generally induces indel mutations within one homology arm because of the NHEJ repair pathway. Therefore, there is a risk of affecting endogenous genes or transgene expression by these indels even if these mutations are controllable in the intron. There is also a risk for the random integration of dsDNA similar to transgenic methods using linear dsDNA. In the F0 founders which we tested, random insertions or complex rearrangements were observed among the Founders (such as *Kcnab1* founders #3 and 4, *Mc4r* founder #7). Random integration might occur anyway when dsDNA donors are used. In our case, cutting circular plasmids by Cas9 inside cells might reduce random integration and the integration of multiple copies of plasmids compared with other methods using linearized dsDNA. NHEJ-mediated mutations or DNA insertions at off-target sites may also eventually occur. A more comprehensive analysis (that is whole-genome sequencing) is required to assess on- and off-target events. Further backcrossing to wild-type animals might segregate such integrations.

In conclusion, the *Combi*-CRISPR method is less time-consuming, easier to prepare, and highly efficient for the generation of KI mice and rats for our tested genes. Donor vector dsDNA, Cas9 protein, and two synthetic sgRNAs can also easily be purchased from custom-order companies.

## Methods

### Animals and zygotes

Iar:Wistar-Imamichi pseudopregnant female rats and Iar:Long-Evans rats (8–10 weeks old) were sourced from Japan SLC, Inc (Hamamatsu, Japan). Iar:Long-Evans cryopreserved zygotes were obtained from the ARK resource (Kumamoto, Japan). Jcl:ICR pseudopregnant female mice and C57BL/6JJcl cryopreserved zygotes were purchased from CLEA Japan Inc (Tokyo, Japan). All animals were housed and maintained under conditions of 50% humidity and a 12:12-h light:dark cycle. They were fed a standard pellet diet (MF, Oriental Yeast Co., Tokyo, Japan) and tap water ad libitum. The Osaka University Animal Experiment Committee approved all animal experiments.

### Preparation of Cas9, sgRNAs, and plasmids

Production and purification of Cas9 mRNA were performed as described previously (Yoshimi et al. 2014, 2016). Cas9 protein was obtained from IDT (Alt-R S.p. Cas9 Nuclease V3, Integrated DNA Technologies, IA, USA). sgRNAs were designed using an online program (https://crispor.tefor.net/) to predict unique target sites throughout the mouse and rat genome. Single-guide RNAs were transcribed in vitro from synthetic double-strand DNAs obtained from IDT or Thermo Fisher Scientific using a MEGAshortscript T7 Transcription Kit (Thermo Fisher Scientific, MA, USA). Specific crRNAs were purchased from IDT (Alt-R CRISPR-Cas9 crRNA) and were assembled with a tracrRNA (Alt-R CRISPR-Cas9 tracrRNA) before use according to the instructions of the manufacturer. Several plasmids used as knock-in donors were purchased from Thermo Fisher Scientific (GeneArt Gene Synthesis). In accordance with the conventional methods, all plasmids were transformed into *Escherichia coli* and extracted with NucleoSpin Plasmid Transfection grade (MACHEREY–NAGEL Gmbh & Co. KG, Germany).

### Microinjections into mouse and rat embryos

Pronuclear-stage mouse embryos were prepared by thawing frozen embryos in KSOM medium (ARK Resource, Kumamoto, Japan) and incubating them for 2–3 h before microinjection. Pronuclear-stage rat embryos were prepared by thawing frozen embryos 2–3 h before microinjection or collecting fresh embryos from naturally mated female rats. Female rats were superovulated by the administration of 150 U/kg of PMSG followed 46–47 h later by 75 U/kg of HCG and mating 1:1 with males. The next day, all females exhibiting copulation plugs were sacrificed and pronuclear embryos were collected from oviducts and maintained under 5% CO_2_ at 37 °C for 2–3 h. All rat embryos were cultured in Rat KSOM medium (ARK Resource). A solution containing 20 ng/µl Cas9 mRNA, 25 ng/µl sgRNA-1, 25 ng/µl sgRNA-2, and 1–3 ng/µl donor plasmid were microinjected into male pronuclei and cytoplasm of mouse embryos using a micromanipulator (Narishige, Tokyo, Japan). Likewise, 100 ng/µl Cas9 protein, 25 ng/µl sgRNA-1, 25 ng/µl sgRNA-2, and 1 ng/µl donor plasmid were microinjected into male pronuclei and cytoplasm of rat embryos. All surviving embryos were transferred on the same day or next day into the oviducts of pseudopregnant surrogate mothers anesthetized with isoflurane (DS Pharma Animal Health Co., Ltd., Osaka, Japan).

### Genotyping analysis

For PCR and sequence analysis, genomic DNA was extracted from a tail biopsy with the KAPA Express Extract DNA Extraction Kit (Kapa Biosystems, London, UK). The sgRNA targeted region, 5′ genome-donor boundary, inside of knock-in donor, and donor-3′ genome boundary were PCR amplified. These PCR amplicons were then directly sequenced using the BigDye Terminator v3.1 cycle sequencing mix and the standard protocol for an Applied Biosystems 3130 DNA Sequencer (Thermo Fisher Scientific). All primer sets used for genotyping analysis are shown in Supplemental Table 2.

## Electronic supplementary material

Below is the link to the electronic supplementary material.

Supplementary file1 (AI 3453 kb)

Supplementary file2 (DOCX 639 kb)

## Data Availability

All data generated or analyzed during this study are included in this published article and its supplementary information files.
