# Supplementary material for: Combi-CRISPR: combination of NHEJ and HDR provides efficient and precise plasmid-based knock-ins in mice and rats
Source: Hum Genet. 2020 Jul 2;140(2):277–87. doi: 10.1007/s00439-020-02198-4 (PMC7864826; doi:10.1007/s00439-020-02198-4)
Supplement: Supplementary file 2 — Supplementary file2 (DOCX 639 kb) [file 439_2020_2198_MOESM2_ESM.docx]

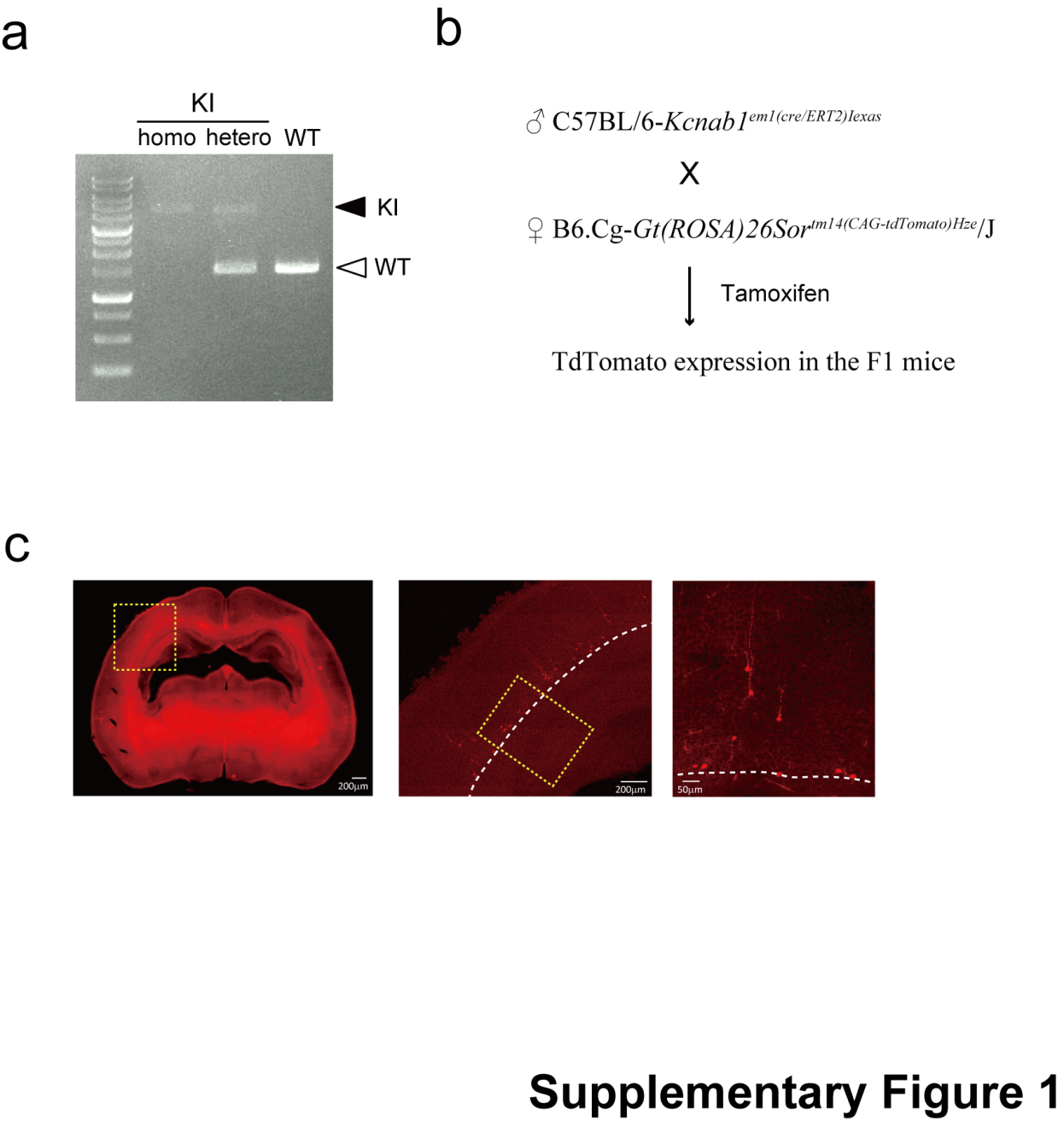


**Supplementary Figure 1. Germline transmission of the KI allele and Cre-induced recombination in the brain of a reporter mouse. a** The germline transmission of the Cre-KI cassette was confirmed as homozygous or heterozygous alleles in F2 mice crossed with wildtype C57BL/6 mice **b** Founder KI mice were crossed with B6.Cg-*Gt(ROSA)26Sor^tm14(CAG-tdTomato)Hze/J^* reporter mice. **c** tdTomato expression was confirmed by the induction of Cre recombination events in the brain neurons of F1 mice.

**Mice**

**Kcnab1 WT**

NNN:TER

NNN:gRNA target

NNN:PAM

NNN:homology arm

NNN:P2A

NNN:iCre

NNN:ERT2

NNN:ins

NNN:del

ctgagccatctctccagccccatgtcttcatttctttagtcaacaaacacaatccctgacttgaagcttatccattgtggggcatggggtagtggatcttcatttcagctatggcttgcaaacatatcgcaagtataaatgactgcttaatgtggactccacaagacaaatgcgcaaccctaaccctctgtgcttctcccctaccctcagGTCCTCCCTAAGATGACATCTCACGTGGTGAACGAGATTGATAACATACTGCGCAACAAGCCCTACAGCAAAAAGGACTATAGATCATAAGGCAATGCATGAGCCACTTAAGCTGCATGGGTAAAGTCATGATGGGTAGGCAGTGCAGAACGGCCTTACCAGCCACTCTTTTCAATCACTTAGCAGTTTGCTGCTCAACCTCTAGTGTCCCAGGATTCTCTGAGGTTTCTGCTGTTGCTACCACTGTGCACCCTGAATTCTGAACACATCTGAAAATTCACAAGCCAGAAAATCCATTCTATTTTCTTATCTTGGACTGGTCCCCTACCCTGGCACTGGTCTGTGTACCTCATGTTTTTGCAATGGAAAGAATGCTGGGGTAGGGGAATCTATTATCTTCAGAAGAAGGCTGTACATATATATTTTTTTCAAAAGAACAAATTTCACAAGATACAGTATATGACCTGCATAGGAAACAGGATATCCTCACTCTTTAGTCTTGCTCGGGAGAACAAACTGAACTAGATTTTTCCCCCCATATTTCATATTCACACTAGCAAGTTTTATGTCACCTGATATTCAAATAAAATTTCTAGATATTTGCTTTCACTATCTCTAAAGTTATATTGTTGCCTGGCCCTGTACAATTTAACTGGGGTGTCTCTCAGGGTTCTAACATATCCATTTGCATAGTTATAAAGGCATGACTATCCCAAGATACATAATAGATACATAATGCTGATTTATAAAAAGGTCACAATGTTATGGAAACAGCTTTAAATAAA

**Kcnab1-ERT2-iCre-ERT2 #1 (+2 bp)**

ctgagccatctctccagccccatgtcttcatttctttagtcaacaaacacaatccctgacttgaagcttatccattgtggggcatggggtagtggatcttcatttcagctatggcttgcaaacatatcgcaagtataaatgactgcttaTTatgtggactccacaagacaaatgcgcaaccctaaccctctgtgcttctcccctaccctcagGTCCTCCCTAAGATGACATCTCACGTGGTGAACGAGATTGATAACATACTGCGCAACAAGCCCTACAGCAAAAAGGACTATAGATCAGGAAGCGGAGCTACTAACTTCAGCCTGCTGAAGCAGGCTGGAGACGTGGAGGAGAACCCTGGACCTATGGCTGGAGACATGAGAGCTGCCAACCTTTGGCCAAGCCCGCTCATGATCAAACGCTCTAAGAAGAACAGCCTGGCCTTGTCCCTGACGGCCGACCAGATGGTCAGTGCCTTGTTGGATGCTGAGCCCCCCATACTCTATTCCGAGTATGATCCTACCAGACCCTTCAGTGAAGCTTCGATGATGGGCTTACTGACCAACCTGGCAGACAGGGAGCTGGTTCACATGATCAACTGGGCGAAGAGGGTGCCAGGCTTTGTGGATTTGACCCTCCATGATCAGGTCCACCTTCTAGAATGTGCCTGGCTAGAGATCCTGATGATTGGTCTCGTCTGGCGCTCCATGGAGCACCCAGTGAAGCTACTGTTTGCTCCTAACTTGCTCTTGGACAGGAACCAGGGAAAATGTGTAGAGGGCATGGTGGAGATCTTCGACATGCTGCTGGCTACATCATCTCGGTTCCGCATGATGAATCTGCAGGGAGAGGAGTTTGTGTGCCTCAAATCTATTATTTTGCTTAATTCTGGAGTGTACACATTTCTGTCCAGCACCCTGAAGTCTCTGGAAGAGAAGGACCATATCCACCGAGTCCTGGACAAGATCACAGACACTTTGATCCACCTGATGGCCAAGGCAGGCCTGACCCTGCAGCAGCAGCACCAGCGGCTGGCCCAGCTCCTCCTCATCCTCTCCCACATCAGGCACATGAGTAACAAAGGCATGGAGCATCTGTACAGCATGAAGTGCAAGAACGTGGTGCCCCTCTATGACCTGCTGCTGGAGGCGGCGGACGCCCACCGCCTACATGCGCCCACTAGCCGTGGAGGGGCATCCGTGGAGGAGACGGACCAAAGCCACTTGGCCACTGCGGGCTCTACTTCATCGCATTCCTTGCAAAAGTATTACATCACGGGGGAGGCAGAGGGTTTCCCTGCCACAGCTGTCGACAACCTGCTGACTGTGCACCAAAACCTGCCTGCCCTCCCTGTGGATGCCACCTCTGATGAAGTCAGGAAGAACCTGATGGACATGTTCAGGGACAGGCAGGCCTTCTCTGAACACACCTGGAAGATGCTCCTGTCTGTGTGCAGATCCTGGGCTGCCTGGTGCAAGCTGAACAACAGGAAATGGTTCCCTGCTGAACCTGAGGATGTGAGGGACTACCTCCTGTACCTGCAAGCCAGAGGCCTGGCTGTGAAGACCATCCAACAGCACCTGGGCCAGCTCAACATGCTGCACAGGAGATCTGGCCTGCCTCGCCCTTCTGACTCCAATGCTGTGTCCCTGGTGATGAGGAGAATCAGAAAGGAGAATGTGGATGCTGGGGAGAGAGCCAAGCAGGCCCTGGCCTTTGAACGCACTGACTTTGACCAAGTCAGATCCCTGATGGAGAACTCTGACAGATGCCAGGACATCAGGAACCTGGCCTTCCTGGGCATTGCCTACAACACCCTGCTGCGCATTGCCGAAATTGCCAGAATCAGAGTGAAGGACATCTCCCGCACCGATGGTGGGAGAATGCTGATCCACATTGGCAGGACCAAGACCCTGGTGTCCACAGCTGGTGTGGAGAAGGCCCTGTCCCTGGGGGTTACCAAGCTGGTGGAGAGATGGATCTCTGTGTCTGGTGTGGCTGATGACCCCAACAACTACCTGTTCTGCCGGGTCAGAAAGAATGGTGTGGCTGCCCCTTCTGCCACCTCCCAACTGTCCACCCGGGCCCTGGAAGGGATCTTTGAGGCCACCCACCGCCTGATCTATGGTGCCAAGGATGACTCTGGGCAGAGATACCTGGCCTGGTCTGGCCACTCTGCCAGAGTGGGTGCTGCCAGGGACATGGCCAGGGCTGGTGTGTCCATCCCTGAAATCATGCAGGCTGGTGGCTGGACCAATGTGAACATtGTGATGAACTACATCAGAAACCTGGACTCTGAGACTGGGGCCATGGTGAGGCTGCTCGAGGATGGGGACCTCGAGCCATCTGCTGGAGACATGAGAGCTGCCAACCTTTGGCCAAGCCCGCTCATGATCAAACGCTCTAAGAAGAACAGCCTGGCCTTGTCCCTGACGGCCGACCAGATGGTCAGTGCCTTGTTGGATGCTGAGCCCCCCATACTCTATTCCGAGTATGATCCTACCAGACCCTTCAGTGAAGCTTCGATGATGGGCTTACTGACCAACCTGGCAGACAGGGAGCTGGTTCACATGATCAACTGGGCGAAGAGGGTGCCAGGCTTTGTGGATTTGACCCTCCATGATCAGGTCCACCTTCTAGAATGTGCCTGGCTAGAGATCCTGATGATTGGTCTCGTCTGGCGCTCCATGGAGCACCCAGTGAAGCTACTGTTTGCTCCTAACTTGCTCTTGGACAGGAACCAGGGAAAATGTGTAGAGGGCATGGTGGAGATCTTCGACATGCTGCTGGCTACATCATCTCGGTTCCGCATGATGAATCTGCAGGGAGAGGAGTTTGTGTGCCTCAAATCTATTATTTTGCTTAATTCTGGAGTGTACACATTTCTGTCCAGCACCCTGAAGTCTCTGGAAGAGAAGGACCATATCCACCGAGTCCTGGACAAGATCACAGACACTTTGATCCACCTGATGGCCAAGGCAGGCCTGACCCTGCAGCAGCAGCACCAGCGGCTGGCCCAGCTCCTCCTCATCCTCTCCCACATCAGGCACATGAGTAACAAAGGCATGGAGCATCTGTACAGCATGAAGTGCAAGAACGTGGTGCCCCTCTATGACCTGCTGCTGGAGGCGGCGGACGCCCACCGCCTACATGCGCCCACTAGCCGTGGAGGGGCATCCGTGGAGGAGACGGACCAAAGCCACTTGGCCACTGCGGGCTCTACTTCATCGCATTCCTTGCAAAAGTATTACATCACGGGGGAGGCAGAGGGTTTCCCTGCCACAGCTTGAGGCAATGCATGAGCCACTTAAGCTGCATGGGTAAAGTCATGATGGGTAGGCAGTGCAGAACGGCCTTACCAGCCACTCTTTTCAATCACTTAGCAGTTTGCTGCTCAACCTCTAGTGTCCCAGGATTCTCTGAGGTTTCTGCTGTTGCTACCACTGTGCACCCTGAATTCTGAACACATCTGAAAATTCACAAGCCAGAAAATCCATTCTATTTTCTTATCTTGGACTGGTCCCCTACCCTGGCACTGGTCTGTGTACCTCATGTTTTTGCAATGGAAAGAATGCTGGGGTAGGGGAATCTATTATCTTCAGAAGAAGGCTGTACATATATATTTTTTTCAAAAGAACAAATTTCACAAGATACAGTATATGACCTGCATAGGAAACAGGATATCCTCACTCTTTAGTCTTGCTCGGGAGAACAAACTGAACTAGATTTTTCCCCCCATATTTCATATTCACACTAGCAAGTTTTATGTCACCTGATATTCAAATAAAATTTCTAGATATTTGCTTTCACTATCTCTAAAGTTATATTGTTGCCTGGCCCTGTACAATTTAACTGGGGTGTCTCTCAGGGTTCTAACATATCCATTTGCATAGTTATAAAGGCATGACTATCCCAAGATACATAATAGATACATAATGCTGATTTATAAAAAGGTCACAATGTTATGGAAACAGCTTTAAATAAA

**Kcnab1-ERT2-iCre-ERT2 #2 (+1 bp)**

ctgagccatctctccagccccatgtcttcatttctttagtcaacaaacacaatccctgacttgaagcttatccattgtggggcatggggtagtggatcttcatttcagctatggcttgcaaacatatcgcaagtataaatgactgcttaTatgtggactccacaagacaaatgcgcaaccctaaccctctgtgcttctcccctaccctcagGTCCTCCCTAAGATGACATCTCACGTGGTGAACGAGATTGATAACATACTGCGCAACAAGCCCTACAGCAAAAAGGACTATAGATCAGGAAGCGGAGCTACTAACTTCAGCCTGCTGAAGCAGGCTGGAGACGTGGAGGAGAACCCTGGACCTATGGCTGGAGACATGAGAGCTGCCAACCTTTGGCCAAGCCCGCTCATGATCAAACGCTCTAAGAAGAACAGCCTGGCCTTGTCCCTGACGGCCGACCAGATGGTCAGTGCCTTGTTGGATGCTGAGCCCCCCATACTCTATTCCGAGTATGATCCTACCAGACCCTTCAGTGAAGCTTCGATGATGGGCTTACTGACCAACCTGGCAGACAGGGAGCTGGTTCACATGATCAACTGGGCGAAGAGGGTGCCAGGCTTTGTGGATTTGACCCTCCATGATCAGGTCCACCTTCTAGAATGTGCCTGGCTAGAGATCCTGATGATTGGTCTCGTCTGGCGCTCCATGGAGCACCCAGTGAAGCTACTGTTTGCTCCTAACTTGCTCTTGGACAGGAACCAGGGAAAATGTGTAGAGGGCATGGTGGAGATCTTCGACATGCTGCTGGCTACATCATCTCGGTTCCGCATGATGAATCTGCAGGGAGAGGAGTTTGTGTGCCTCAAATCTATTATTTTGCTTAATTCTGGAGTGTACACATTTCTGTCCAGCACCCTGAAGTCTCTGGAAGAGAAGGACCATATCCACCGAGTCCTGGACAAGATCACAGACACTTTGATCCACCTGATGGCCAAGGCAGGCCTGACCCTGCAGCAGCAGCACCAGCGGCTGGCCCAGCTCCTCCTCATCCTCTCCCACATCAGGCACATGAGTAACAAAGGCATGGAGCATCTGTACAGCATGAAGTGCAAGAACGTGGTGCCCCTCTATGACCTGCTGCTGGAGGCGGCGGACGCCCACCGCCTACATGCGCCCACTAGCCGTGGAGGGGCATCCGTGGAGGAGACGGACCAAAGCCACTTGGCCACTGCGGGCTCTACTTCATCGCATTCCTTGCAAAAGTATTACATCACGGGGGAGGCAGAGGGTTTCCCTGCCACAGCTGTCGACAACCTGCTGACTGTGCACCAAAACCTGCCTGCCCTCCCTGTGGATGCCACCTCTGATGAAGTCAGGAAGAACCTGATGGACATGTTCAGGGACAGGCAGGCCTTCTCTGAACACACCTGGAAGATGCTCCTGTCTGTGTGCAGATCCTGGGCTGCCTGGTGCAAGCTGAACAACAGGAAATGGTTCCCTGCTGAACCTGAGGATGTGAGGGACTACCTCCTGTACCTGCAAGCCAGAGGCCTGGCTGTGAAGACCATCCAACAGCACCTGGGCCAGCTCAACATGCTGCACAGGAGATCTGGCCTGCCTCGCCCTTCTGACTCCAATGCTGTGTCCCTGGTGATGAGGAGAATCAGAAAGGAGAATGTGGATGCTGGGGAGAGAGCCAAGCAGGCCCTGGCCTTTGAACGCACTGACTTTGACCAAGTCAGATCCCTGATGGAGAACTCTGACAGATGCCAGGACATCAGGAACCTGGCCTTCCTGGGCATTGCCTACAACACCCTGCTGCGCATTGCCGAAATTGCCAGAATCAGAGTGAAGGACATCTCCCGCACCGATGGTGGGAGAATGCTGATCCACATTGGCAGGACCAAGACCCTGGTGTCCACAGCTGGTGTGGAGAAGGCCCTGTCCCTGGGGGTTACCAAGCTGGTGGAGAGATGGATCTCTGTGTCTGGTGTGGCTGATGACCCCAACAACTACCTGTTCTGCCGGGTCAGAAAGAATGGTGTGGCTGCCCCTTCTGCCACCTCCCAACTGTCCACCCGGGCCCTGGAAGGGATCTTTGAGGCCACCCACCGCCTGATCTATGGTGCCAAGGATGACTCTGGGCAGAGATACCTGGCCTGGTCTGGCCACTCTGCCAGAGTGGGTGCTGCCAGGGACATGGCCAGGGCTGGTGTGTCCATCCCTGAAATCATGCAGGCTGGTGGCTGGACCAATGTGAACATtGTGATGAACTACATCAGAAACCTGGACTCTGAGACTGGGGCCATGGTGAGGCTGCTCGAGGATGGGGACCTCGAGCCATCTGCTGGAGACATGAGAGCTGCCAACCTTTGGCCAAGCCCGCTCATGATCAAACGCTCTAAGAAGAACAGCCTGGCCTTGTCCCTGACGGCCGACCAGATGGTCAGTGCCTTGTTGGATGCTGAGCCCCCCATACTCTATTCCGAGTATGATCCTACCAGACCCTTCAGTGAAGCTTCGATGATGGGCTTACTGACCAACCTGGCAGACAGGGAGCTGGTTCACATGATCAACTGGGCGAAGAGGGTGCCAGGCTTTGTGGATTTGACCCTCCATGATCAGGTCCACCTTCTAGAATGTGCCTGGCTAGAGATCCTGATGATTGGTCTCGTCTGGCGCTCCATGGAGCACCCAGTGAAGCTACTGTTTGCTCCTAACTTGCTCTTGGACAGGAACCAGGGAAAATGTGTAGAGGGCATGGTGGAGATCTTCGACATGCTGCTGGCTACATCATCTCGGTTCCGCATGATGAATCTGCAGGGAGAGGAGTTTGTGTGCCTCAAATCTATTATTTTGCTTAATTCTGGAGTGTACACATTTCTGTCCAGCACCCTGAAGTCTCTGGAAGAGAAGGACCATATCCACCGAGTCCTGGACAAGATCACAGACACTTTGATCCACCTGATGGCCAAGGCAGGCCTGACCCTGCAGCAGCAGCACCAGCGGCTGGCCCAGCTCCTCCTCATCCTCTCCCACATCAGGCACATGAGTAACAAAGGCATGGAGCATCTGTACAGCATGAAGTGCAAGAACGTGGTGCCCCTCTATGACCTGCTGCTGGAGGCGGCGGACGCCCACCGCCTACATGCGCCCACTAGCCGTGGAGGGGCATCCGTGGAGGAGACGGACCAAAGCCACTTGGCCACTGCGGGCTCTACTTCATCGCATTCCTTGCAAAAGTATTACATCACGGGGGAGGCAGAGGGTTTCCCTGCCACAGCTTGAGGCAATGCATGAGCCACTTAAGCTGCATGGGTAAAGTCATGATGGGTAGGCAGTGCAGAACGGCCTTACCAGCCACTCTTTTCAATCACTTAGCAGTTTGCTGCTCAACCTCTAGTGTCCCAGGATTCTCTGAGGTTTCTGCTGTTGCTACCACTGTGCACCCTGAATTCTGAACACATCTGAAAATTCACAAGCCAGAAAATCCATTCTATTTTCTTATCTTGGACTGGTCCCCTACCCTGGCACTGGTCTGTGTACCTCATGTTTTTGCAATGGAAAGAATGCTGGGGTAGGGGAATCTATTATCTTCAGAAGAAGGCTGTACATATATATTTTTTTCAAAAGAACAAATTTCACAAGATACAGTATATGACCTGCATAGGAAACAGGATATCCTCACTCTTTAGTCTTGCTCGGGAGAACAAACTGAACTAGATTTTTCCCCCCATATTTCATATTCACACTAGCAAGTTTTATGTCACCTGATATTCAAATAAAATTTCTAGATATTTGCTTTCACTATCTCTAAAGTTATATTGTTGCCTGGCCCTGTACAATTTAACTGGGGTGTCTCTCAGGGTTCTAACATATCCATTTGCATAGTTATAAAGGCATGACTATCCCAAGATACATAATAGATACATAATGCTGATTTATAAAAAGGTCACAATGTTATGGAAACAGCTTTAAATAAA

**Kcnab1-ERT2-iCre-ERT2 #5 (-57 bp)**

ctgagccatctctccagccccatgtcttcatttctttagtcaacaaacacaatccctgacttgaagcttatccattgtggggcatggggtagtggatcttcatttcagctatggcttgcaaacatatcgcaagtataaatgactgcttaatgtggactccacaagacaaatgcgcaaccctaaccctctgtgcttctcccctaccctcagGTCCTCCCTAAGATGACATCTCACGTGGTGAACGAGATTGATAACATACTGCGCAACAAGCCCTACAGCAAAAAGGACTATAGATCAGGAAGCGGAGCTACTAACTTCAGCCTGCTGAAGCAGGCTGGAGACGTGGAGGAGAACCCTGGACCTATGGCTGGAGACATGAGAGCTGCCAACCTTTGGCCAAGCCCGCTCATGATCAAACGCTCTAAGAAGAACAGCCTGGCCTTGTCCCTGACGGCCGACCAGATGGTCAGTGCCTTGTTGGATGCTGAGCCCCCCATACTCTATTCCGAGTATGATCCTACCAGACCCTTCAGTGAAGCTTCGATGATGGGCTTACTGACCAACCTGGCAGACAGGGAGCTGGTTCACATGATCAACTGGGCGAAGAGGGTGCCAGGCTTTGTGGATTTGACCCTCCATGATCAGGTCCACCTTCTAGAATGTGCCTGGCTAGAGATCCTGATGATTGGTCTCGTCTGGCGCTCCATGGAGCACCCAGTGAAGCTACTGTTTGCTCCTAACTTGCTCTTGGACAGGAACCAGGGAAAATGTGTAGAGGGCATGGTGGAGATCTTCGACATGCTGCTGGCTACATCATCTCGGTTCCGCATGATGAATCTGCAGGGAGAGGAGTTTGTGTGCCTCAAATCTATTATTTTGCTTAATTCTGGAGTGTACACATTTCTGTCCAGCACCCTGAAGTCTCTGGAAGAGAAGGACCATATCCACCGAGTCCTGGACAAGATCACAGACACTTTGATCCACCTGATGGCCAAGGCAGGCCTGACCCTGCAGCAGCAGCACCAGCGGCTGGCCCAGCTCCTCCTCATCCTCTCCCACATCAGGCACATGAGTAACAAAGGCATGGAGCATCTGTACAGCATGAAGTGCAAGAACGTGGTGCCCCTCTATGACCTGCTGCTGGAGGCGGCGGACGCCCACCGCCTACATGCGCCCACTAGCCGTGGAGGGGCATCCGTGGAGGAGACGGACCAAAGCCACTTGGCCACTGCGGGCTCTACTTCATCGCATTCCTTGCAAAAGTATTACATCACGGGGGAGGCAGAGGGTTTCCCTGCCACAGCTGTCGACAACCTGCTGACTGTGCACCAAAACCTGCCTGCCCTCCCTGTGGATGCCACCTCTGATGAAGTCAGGAAGAACCTGATGGACATGTTCAGGGACAGGCAGGCCTTCTCTGAACACACCTGGAAGATGCTCCTGTCTGTGTGCAGATCCTGGGCTGCCTGGTGCAAGCTGAACAACAGGAAATGGTTCCCTGCTGAACCTGAGGATGTGAGGGACTACCTCCTGTACCTGCAAGCCAGAGGCCTGGCTGTGAAGACCATCCAACAGCACCTGGGCCAGCTCAACATGCTGCACAGGAGATCTGGCCTGCCTCGCCCTTCTGACTCCAATGCTGTGTCCCTGGTGATGAGGAGAATCAGAAAGGAGAATGTGGATGCTGGGGAGAGAGCCAAGCAGGCCCTGGCCTTTGAACGCACTGACTTTGACCAAGTCAGATCCCTGATGGAGAACTCTGACAGATGCCAGGACATCAGGAACCTGGCCTTCCTGGGCATTGCCTACAACACCCTGCTGCGCATTGCCGAAATTGCCAGAATCAGAGTGAAGGACATCTCCCGCACCGATGGTGGGAGAATGCTGATCCACATTGGCAGGACCAAGACCCTGGTGTCCACAGCTGGTGTGGAGAAGGCCCTGTCCCTGGGGGTTACCAAGCTGGTGGAGAGATGGATCTCTGTGTCTGGTGTGGCTGATGACCCCAACAACTACCTGTTCTGCCGGGTCAGAAAGAATGGTGTGGCTGCCCCTTCTGCCACCTCCCAACTGTCCACCCGGGCCCTGGAAGGGATCTTTGAGGCCACCCACCGCCTGATCTATGGTGCCAAGGATGACTCTGGGCAGAGATACCTGGCCTGGTCTGGCCACTCTGCCAGAGTGGGTGCTGCCAGGGACATGGCCAGGGCTGGTGTGTCCATCCCTGAAATCATGCAGGCTGGTGGCTGGACCAATGTGAACATtGTGATGAACTACATCAGAAACCTGGACTCTGAGACTGGGGCCATGGTGAGGCTGCTCGAGGATGGGGACCTCGAGCCATCTGCTGGAGACATGAGAGCTGCCAACCTTTGGCCAAGCCCGCTCATGATCAAACGCTCTAAGAAGAACAGCCTGGCCTTGTCCCTGACGGCCGACCAGATGGTCAGTGCCTTGTTGGATGCTGAGCCCCCCATACTCTATTCCGAGTATGATCCTACCAGACCCTTCAGTGAAGCTTCGATGATGGGCTTACTGACCAACCTGGCAGACAGGGAGCTGGTTCACATGATCAACTGGGCGAAGAGGGTGCCAGGCTTTGTGGATTTGACCCTCCATGATCAGGTCCACCTTCTAGAATGTGCCTGGCTAGAGATCCTGATGATTGGTCTCGTCTGGCGCTCCATGGAGCACCCAGTGAAGCTACTGTTTGCTCCTAACTTGCTCTTGGACAGGAACCAGGGAAAATGTGTAGAGGGCATGGTGGAGATCTTCGACATGCTGCTGGCTACATCATCTCGGTTCCGCATGATGAATCTGCAGGGAGAGGAGTTTGTGTGCCTCAAATCTATTATTTTGCTTAATTCTGGAGTGTACACATTTCTGTCCAGCACCCTGAAGTCTCTGGAAGAGAAGGACCATATCCACCGAGTCCTGGACAAGATCACAGACACTTTGATCCACCTGATGGCCAAGGCAGGCCTGACCCTGCAGCAGCAGCACCAGCGGCTGGCCCAGCTCCTCCTCATCCTCTCCCACATCAGGCACATGAGTAACAAAGGCATGGAGCATCTGTACAGCATGAAGTGCAAGAACGTGGTGCCCCTCTATGACCTGCTGCTGGAGGCGGCGGACGCCCACCGCCTACATGCGCCCACTAGCCGTGGAGGGGCATCCGTGGAGGAGACGGACCAAAGCCACTTGGCCACTGCGGGCTCTACTTCATCGCATTCCTTGCAAAAGTATTACATCACGGGGGAGGCAGAGGGTTTCCCTGCCACAGCTTGAGGCAATGCATGAGCCACTTAAGCTGCATGGGTAAAGTCATGATGGGTAGGCAGTGCAGAACGGCCTTACCAGCCACTCTTTTCAATCACTTAGCAGTTTGCTGCTCAACCTCTAGTGTCCCAGGATTCTCTGAGGTTTCTGCTGTTGCTACCACTGTGCACCCTGAATTCTGAACACATCTGAAAATTCACAAGCCAGAAAATCCATTCTATTTTCTTATCTTGGACTGGTCCCCTACCCTGGCACTGGTCTGTGTACCTCATGTTTTTGCAATGGAAAGAATGCTGGGGTAGGGGAATCTATTATCTTCAGAAGAAGGCTGTACATATATATTTTTTTCAAAAGAACAAATTTCACAAGATACAGTATATGACCTGCATAGGAAACAGGATATCCTCACTCTTTAGTCTTGCTCGGGAGAACAAACTGAACTAGATTTTTCCCCCCATATTTCATATTCACACTAGCAAGTTTTATGTCACCTGATATTCAAATAAAATTTCTAGATATTTGCTTTCACTATCTCTAAAGTTATATTGTTGCCTGGCCCTGTACAATTTAACTGGGGTGTCTCTCAGGGTTCTAACATATCCATTTGCATAGTTATAAAGGCATGACTATCCCAAGATACATAATAGATACATAATGCTGATTTATAAAAAGGTCACAATGTTATGGAAACAGCTTTAAATAAA

**Mc4r WT**

NNN:TER

NNN:gRNA target

NNN:PAM

NNN:homology arm

NNN:T2A

NNN:iCre

NNN:del

NNN:ins

GTTGGAGAACATTCTAGTGATCGTGGCGATAGCCAAGAACAAGAACCTGCACTCACCCATGTACTTTTTCATCTGTAGCCTGGCTGTGGCAGATATGCTGGTGAGCGTTTCGAATGGGTCGGAAACCATCGTCATTACCCTGTTAAACAGTACGGATACGGATGCCCAGAGCTTCACCGTGAACATTGATAATGTCATTGACTCTGTGATCTGTAGCTCCTTGCTCGCATCCATTTGCAGCCTGCTTTCCATTGCGGTGGACAGGTATTTCACTATCTTTTACGCGCTCCAGTACCATAACATCATGACGGTTAGGCGGGTCGGGATCATCATAAGTTGTATCTGGGCAGCTTGCACTGTGTCAGGCGTCCTCTTCATCATTTACTCGGACAGCAGCGCTGTCATCATCTGCCTCATTTCCATGTTCTTCACTATGCTAGTTCTCATGGCCTCTCTCTATGTCCACATGTTCCTGATGGCGAGGCTTCACATTAAGAGGATTGCTGTCCTCCCAGGCACAGGGACCATCCGCCAGGGTACCAACATGAAGGGGGCGATTACCTTGACCATCCTGATTGGAGTCTTTGTTGTCTGCTGGGCCCCGTTCTTTCTCCATTTACTGTTCTACATCTCTTGCCCTCAGAATCCATACTGCGTGTGCTTCATGTCTCATTTTAATTTGTATCTCATACTGATCATGTGTAACGCCGTCATCGACCCTCTCATTTATGCCCTCCGGAGTCAAGAACTGAGGAAAACTTTCAAAGAGATCATCTGTTTCTATCCTCTGGGAGGCATCTGTGAGTTGTCTAGCAGGTATTAAGTGGGGGACAGAGTGCAAACTAGGTAGATACCTGCAGACTTTGTCACTCTGGCCCGATCTGAGCAGTGTACTTCCCAACAGCTGCCTCTTCTGTGTAATGCTTTGGTTGAAAATATCTACTGTATAAATGTAAGTTTGTGACTTTTGACATGGAAAAAAAAGTCTCAACGTGTTATGTTTATTGACACGCTATTTTTTTTGTTTGTAAAATGCTTATTTATGTTCTATATAGTGTGGGCGTTATGAATTGACATGAAAGAAAAACAGACACCCTTATTAAAACTTTGACAGTGTTTCTTTCCTGTTATTTATCAAGGTTCCACACTTGTTCTTTCTGTAGTGGCCGAAATCAGAACCTTATTAAACGTGTTCTCAGCTGTTCTCATGTATTAGCCCCACAGTACTGCAGAGGCACTGACCCCACTGTTTATGGGGAAATATTTAAACACTACATGCTTGATCATTAAAATGAGTCAGCTCTCTTAGTGAAATTTCGAGCAATCGAATAAAAGCTTGCCTATTATCCTTGCTGTCCAAATACACTGATGCTTCTTTTTAAGTAAAGGAAAGAGAAAGGGGGAAGAAGCAGCTACTGAGGAGAAAGTGAGATTTCTGTCACATGCATTTCTCCAAGAAGGAATGGTTCATTGCCCGAGACTCAGAGTTCACACAGGCAAGTCAGCTGTGGTAGGGGAAATGC

**Mc4r-iCre #2 (-3 bp)**

GTTGGAGAACATTCTAGTGATCGTGGCGATAGCCAAGAACAAGAACCTGCACTCACCCATGTACTTTTTCATCTGTAGCCTGGCTGTGGCAGATATGCTGGTGAGCGTTTCGAATGGGTCGGAAACCATCGTCATTACCCTGTTAAACAGTACGGATACGGATGCCCAGAGCTTCACCGTGAACATTGATAATGTCATTGACTCTGTGATCTGTAGCTCCTTGCTCGCATCCATTTGCAGCCTGCTTTCCATTGCGGTGGACAGGTATTTCACTATCTTTTACGCGCTCCAGTACCATAACATCATGACGGTTAGGCGGGTCGGGATCATCATAAGTTGTATCTGGGCAGCTTGCACTGTGTCAGGCGTCCTCTTCATCATTTACTCGGACAGCAGCGCTGTCATCATCTGCCTCATTTCCATGTTCTTCACTATGCTAGTTCTCATGGCCTCTCTCTATGTCCACATGTTCCTGATGGCGAGGCTTCACATTAAGAGGATTGCTGTCCTCCCAGGCACAGGGACCATCCGCCAGGGTACCAACATGAAGGGGGCGATTACCTTGACCATCCTGATTGGAGTCTTTGTTGTCTGCTGGGCCCCGTTCTTTCTCCATTTACTGTTCTACATCTCTTGCCCTCAGAATCCATACTGCGTGTGCTTCATGTCTCATTTTAATTTGTATCTCATACTGATCATGTGTAACGCCGTCATCGACCCTCTCATTTATGCCCTCCGGAGTCAAGAACTGAGGAAAACTTTCAAAGAGATCATCTGTTTCTATCCTCTGGGAGGCATCTGTGAGTTGTCTAGCAGGTATGAGGGCAGAGGAAGTCTTCTAACATGCGGTGACGTGGAGGAGAATCCCGGCCCTATGGTGCCCAAGAAGAAGAGGAAAGTCTCCAACCTGCTGACTGTGCACCAAAACCTGCCTGCCCTCCCTGTGGATGCCACCTCTGATGAAGTCAGGAAGAACCTGATGGACATGTTCAGGGACAGGCAGGCCTTCTCTGAACACACCTGGAAGATGCTCCTGTCTGTGTGCAGATCCTGGGCTGCCTGGTGCAAGCTGAACAACAGGAAATGGTTCCCTGCTGAACCTGAGGATGTGAGGGACTACCTCCTGTACCTGCAAGCCAGAGGCCTGGCTGTGAAGACCATCCAACAGCACCTGGGCCAGCTCAACATGCTGCACAGGAGATCTGGCCTGCCTCGCCCTTCTGACTCCAATGCTGTGTCCCTGGTGATGAGGAGAATCAGAAAGGAGAATGTGGATGCTGGGGAGAGAGCCAAGCAGGCCCTGGCCTTTGAACGCACTGACTTTGACCAAGTCAGATCCCTGATGGAGAACTCTGACAGATGCCAGGACATCAGGAACCTGGCCTTCCTGGGCATTGCCTACAACACCCTGCTGCGCATTGCCGAAATTGCCAGAATCAGAGTGAAGGACATCTCCCGCACCGATGGTGGGAGAATGCTGATCCACATTGGCAGGACCAAGACCCTGGTGTCCACAGCTGGTGTGGAGAAGGCCCTGTCCCTGGGGGTTACCAAGCTGGTGGAGAGATGGATCTCTGTGTCTGGTGTGGCTGATGACCCCAACAACTACCTGTTCTGCCGGGTCAGAAAGAATGGTGTGGCTGCCCCTTCTGCCACCTCCCAACTGTCCACCCGGGCCCTGGAAGGGATCTTTGAGGCCACCCACCGCCTGATCTATGGTGCCAAGGATGACTCTGGGCAGAGATACCTGGCCTGGTCTGGCCACTCTGCCAGAGTGGGTGCTGCCAGGGACATGGCCAGGGCTGGTGTGTCCATCCCTGAAATCATGCAGGCTGGTGGCTGGACCAATGTGAACATTGTGATGAACTACATCAGAAACCTGGACTCTGAGACTGGGGCCATGGTGAGGCTGCTCGAGGATGGGGACTAAGTGGGGGACAGAGTGCAAACTAGGTAGATACCTGCAGACTTTGTCACTCTGGCCCGATCTGAGCAGTGTACTTCCCAACAGCTGCCTCTTCTGTGTAATGCTTTGGTTGAAAATATCTACTGTATAAATGTAAGTTTGTGACTTTTGACATGGAAAAAAAAGTCTCAACGTGTTATGTTTATTGACACGCTATTTTTTTTGTTTGTAAAATGCTTATTTATGTTCTATATAGTGTGGGCGTTATGAATTGACATGAAAGAAAAACAGACACCCTTATTAAAACTTTGACAGTGTTTCTTTCCTGTTATTTATCAAGGTTCCACACTTGTTCTTTCTGTAGTGGCCGAAATCAGAACCTTATTAAACGTGTTCTCAGCTGTTCTCATGTATTAGCCCCACAGTACTGCAGAGGCACTGACCCCACTGTTTATGGGGAAATATTTAAACACTACATGCTTGATCATTAAAATGAGTCAGCTCTCTTAGTGAAATTTCGAGCAATCGAATAAAAGCTTGCCTATTATCCTTGCTGTCCAAATACACTGATGCTTCTTTTTAAGTAAAGGAAAGAGAAAGGGGGAAGAAGCAGCTACTGAGGAGAAAGTGAGATTTCTGTCACATGCATTTCTCCAAGAAGGAATGGTTCATTGCCCGAGACTCAGAGTTCACACAGGCAAGTCAGCTGTGGTAGGGGAAATGC

**Mc4r-iCre #3 (-86 bp and +3 bp)**

GTTGGAGAACATTCTAGTGATCGTGGCGATAGCCAAGAACAAGAACCTGCACTCACCCATGTACTTTTTCATCTGTAGCCTGGCTGTGGCAGATATGCTGGTGAGCGTTTCGAATGGGTCGGAAACCATCGTCATTACCCTGTTAAACAGTACGGATACGGATGCCCAGAGCTTCACCGTGAACATTGATAATGTCATTGACTCTGTGATCTGTAGCTCCTTGCTCGCATCCATTTGCAGCCTGCTTTCCATTGCGGTGGACAGGTATTTCACTATCTTTTACGCGCTCCAGTACCATAACATCATGACGGTTAGGCGGGTCGGGATCATCATAAGTTGTATCTGGGCAGCTTGCACTGTGTCAGGCGTCCTCTTCATCATTTACTCGGACAGCAGCGCTGTCATCATCTGCCTCATTTCCATGTTCTTCACTATGCTAGTTCTCATGGCCTCTCTCTATGTCCACATGTTCCTGATGGCGAGGCTTCACATTAAGAGGATTGCTGTCCTCCCAGGCACAGGGACCATCCGCCAGGGTACCAACATGAAGGGGGCGATTACCTTGACCATCCTGATTGGAGTCTTTGTTGTCTGCTGGGCCCCGTTCTTTCTCCATTTACTGTTCTACATCTCTTGCCCTCAGAATCCATACTGCGTGTGCTTCATGTCTCATTTTAATTTGTATCTCATACTGATCATGTGTAACGCCGTCATCGACCCTCTCATTTATGCCCTCCGGAGTCAAGAACTGAGGAAAACTTTCAAAGAGATCATCTGTTTCTATCCTCTGGGAGGCATCTGTGAGTTGTCTAGCAGGTATGAGGGCAGAGGAAGTCTTCTAACATGCGGTGACGTGGAGGAGAATCCCGGCCCTATGGTGCCCAAGAAGAAGAGGAAAGTCTCCAACCTGCTGACTGTGCACCAAAACCTGCCTGCCCTCCCTGTGGATGCCACCTCTGATGAAGTCAGGAAGAACCTGATGGACATGTTCAGGGACAGGCAGGCCTTCTCTGAACACACCTGGAAGATGCTCCTGTCTGTGTGCAGATCCTGGGCTGCCTGGTGCAAGCTGAACAACAGGAAATGGTTCCCTGCTGAACCTGAGGATGTGAGGGACTACCTCCTGTACCTGCAAGCCAGAGGCCTGGCTGTGAAGACCATCCAACAGCACCTGGGCCAGCTCAACATGCTGCACAGGAGATCTGGCCTGCCTCGCCCTTCTGACTCCAATGCTGTGTCCCTGGTGATGAGGAGAATCAGAAAGGAGAATGTGGATGCTGGGGAGAGAGCCAAGCAGGCCCTGGCCTTTGAACGCACTGACTTTGACCAAGTCAGATCCCTGATGGAGAACTCTGACAGATGCCAGGACATCAGGAACCTGGCCTTCCTGGGCATTGCCTACAACACCCTGCTGCGCATTGCCGAAATTGCCAGAATCAGAGTGAAGGACATCTCCCGCACCGATGGTGGGAGAATGCTGATCCACATTGGCAGGACCAAGACCCTGGTGTCCACAGCTGGTGTGGAGAAGGCCCTGTCCCTGGGGGTTACCAAGCTGGTGGAGAGATGGATCTCTGTGTCTGGTGTGGCTGATGACCCCAACAACTACCTGTTCTGCCGGGTCAGAAAGAATGGTGTGGCTGCCCCTTCTGCCACCTCCCAACTGTCCACCCGGGCCCTGGAAGGGATCTTTGAGGCCACCCACCGCCTGATCTATGGTGCCAAGGATGACTCTGGGCAGAGATACCTGGCCTGGTCTGGCCACTCTGCCAGAGTGGGTGCTGCCAGGGACATGGCCAGGGCTGGTGTGTCCATCCCTGAAATCATGCAGGCTGGTGGCTGGACCAATGTGAACATTGTGATGAACTACATCAGAAACCTGGACTCTGAGACTGGGGCCATGGTGAGGCTGCTCGAGGATGGGGACTAAGTGGGGGACAGAGTGCAAACTAGGTAGATACCTGCAGACTTTGTCACTCTGGCCCGATCTGAGCAGTGTACTTCCCAACAGCTGCCTCTTCTGTGTAATGCTTTGGTTGAAAATATCTACTGTATAAATGTAAGTTTGTGACTTTTGACATGGAAAAAAAAGTCTCAACGTGTTATGTTTATTGACACGCTATTTTTTTTGTTTGTAAAATGCTTATTTATGTTCTATATAGTGTGGGCGTTATGAATTGACATGAAAGAAAAACAGACACCCTTATTAAAACTTTGACAGTGTTTCTTTCCTGTTATTTATCAAGGTTCCCTGACACTTGTTCTTTCTGTAGTGGCCGAAATCAGAACCTTATTAAACGTGTTCTCAGCTGTTCTCATGTATTAGCCCCACAGTACTGCAGAGGCACTGACCCCACTGTTTATGGGGAAATATTTAAACACTACATGCTTGATCATTAAAATGAGTCAGCTCTCTTAGTGAAATTTCGAGCAATCGAATAAAAGCTTGCCTATTATCCTTGCTGTCCAAATACACTGATGCTTCTTTTTAAGTAAAGGAAAGAGAAAGGGGGAAGAAGCAGCTACTGAGGAGAAAGTGAGATTTCTGTCACATGCATTTCTCCAAGAAGGAATGGTTCATTGCCCGAGACTCAGAGTTCACACAGGCAAGTCAGCTGTGGTAGGGGAAATGC

**Mc4r-iCre #4 (-4 bp)**

GTTGGAGAACATTCTAGTGATCGTGGCGATAGCCAAGAACAAGAACCTGCACTCACCCATGTACTTTTTCATCTGTAGCCTGGCTGTGGCAGATATGCTGGTGAGCGTTTCGAATGGGTCGGAAACCATCGTCATTACCCTGTTAAACAGTACGGATACGGATGCCCAGAGCTTCACCGTGAACATTGATAATGTCATTGACTCTGTGATCTGTAGCTCCTTGCTCGCATCCATTTGCAGCCTGCTTTCCATTGCGGTGGACAGGTATTTCACTATCTTTTACGCGCTCCAGTACCATAACATCATGACGGTTAGGCGGGTCGGGATCATCATAAGTTGTATCTGGGCAGCTTGCACTGTGTCAGGCGTCCTCTTCATCATTTACTCGGACAGCAGCGCTGTCATCATCTGCCTCATTTCCATGTTCTTCACTATGCTAGTTCTCATGGCCTCTCTCTATGTCCACATGTTCCTGATGGCGAGGCTTCACATTAAGAGGATTGCTGTCCTCCCAGGCACAGGGACCATCCGCCAGGGTACCAACATGAAGGGGGCGATTACCTTGACCATCCTGATTGGAGTCTTTGTTGTCTGCTGGGCCCCGTTCTTTCTCCATTTACTGTTCTACATCTCTTGCCCTCAGAATCCATACTGCGTGTGCTTCATGTCTCATTTTAATTTGTATCTCATACTGATCATGTGTAACGCCGTCATCGACCCTCTCATTTATGCCCTCCGGAGTCAAGAACTGAGGAAAACTTTCAAAGAGATCATCTGTTTCTATCCTCTGGGAGGCATCTGTGAGTTGTCTAGCAGGTATGAGGGCAGAGGAAGTCTTCTAACATGCGGTGACGTGGAGGAGAATCCCGGCCCTATGGTGCCCAAGAAGAAGAGGAAAGTCTCCAACCTGCTGACTGTGCACCAAAACCTGCCTGCCCTCCCTGTGGATGCCACCTCTGATGAAGTCAGGAAGAACCTGATGGACATGTTCAGGGACAGGCAGGCCTTCTCTGAACACACCTGGAAGATGCTCCTGTCTGTGTGCAGATCCTGGGCTGCCTGGTGCAAGCTGAACAACAGGAAATGGTTCCCTGCTGAACCTGAGGATGTGAGGGACTACCTCCTGTACCTGCAAGCCAGAGGCCTGGCTGTGAAGACCATCCAACAGCACCTGGGCCAGCTCAACATGCTGCACAGGAGATCTGGCCTGCCTCGCCCTTCTGACTCCAATGCTGTGTCCCTGGTGATGAGGAGAATCAGAAAGGAGAATGTGGATGCTGGGGAGAGAGCCAAGCAGGCCCTGGCCTTTGAACGCACTGACTTTGACCAAGTCAGATCCCTGATGGAGAACTCTGACAGATGCCAGGACATCAGGAACCTGGCCTTCCTGGGCATTGCCTACAACACCCTGCTGCGCATTGCCGAAATTGCCAGAATCAGAGTGAAGGACATCTCCCGCACCGATGGTGGGAGAATGCTGATCCACATTGGCAGGACCAAGACCCTGGTGTCCACAGCTGGTGTGGAGAAGGCCCTGTCCCTGGGGGTTACCAAGCTGGTGGAGAGATGGATCTCTGTGTCTGGTGTGGCTGATGACCCCAACAACTACCTGTTCTGCCGGGTCAGAAAGAATGGTGTGGCTGCCCCTTCTGCCACCTCCCAACTGTCCACCCGGGCCCTGGAAGGGATCTTTGAGGCCACCCACCGCCTGATCTATGGTGCCAAGGATGACTCTGGGCAGAGATACCTGGCCTGGTCTGGCCACTCTGCCAGAGTGGGTGCTGCCAGGGACATGGCCAGGGCTGGTGTGTCCATCCCTGAAATCATGCAGGCTGGTGGCTGGACCAATGTGAACATTGTGATGAACTACATCAGAAACCTGGACTCTGAGACTGGGGCCATGGTGAGGCTGCTCGAGGATGGGGACTAAGTGGGGGACAGAGTGCAAACTAGGTAGATACCTGCAGACTTTGTCACTCTGGCCCGATCTGAGCAGTGTACTTCCCAACAGCTGCCTCTTCTGTGTAATGCTTTGGTTGAAAATATCTACTGTATAAATGTAAGTTTGTGACTTTTGACATGGAAAAAAAAGTCTCAACGTGTTATGTTTATTGACACGCTATTTTTTTTGTTTGTAAAATGCTTATTTATGTTCTATATAGTGTGGGCGTTATGAATTGACATGAAAGAAAAACAGACACCCTTATTAAAACTTTGACAGTGTTTCTTTCCTGTTATTTATCAAGGTTCCACACTTGTTCTTTCTGTAGTGGCCGAAATCAGAACCTTATTAAACGTGTTCTCAGCTGTTCTCATGTATTAGCCCCACAGTACTGCAGAGGCACTGACCCCACTGTTTATGGGGAAATATTTAAACACTACATGCTTGATCATTAAAATGAGTCAGCTCTCTTAGTGAAATTTCGAGCAATCGAATAAAAGCTTGCCTATTATCCTTGCTGTCCAAATACACTGATGCTTCTTTTTAAGTAAAGGAAAGAGAAAGGGGGAAGAAGCAGCTACTGAGGAGAAAGTGAGATTTCTGTCACATGCATTTCTCCAAGAAGGAATGGTTCATTGCCCGAGACTCAGAGTTCACACAGGCAAGTCAGCTGTGGTAGGGGAAATGC

**Slc12a1 WT**

NNN:TER

NNN:gRNA target

NNN:PAM

NNN:homology arm

NNN:T2A

NNN:iCre

NNN:del

GAGCTGTGTATGTCCGTGGCTAACTCTGTACAGTGTACAGTGTGGGAACAGGTCAACAAAGAAAAAAGGCCACGAGCTTTTCCATTTTCATTTCTTATCGCATGGCCCTACCAATGTTAGGAAAATACCTACTGAAAATATTACTCATGAAATGAAGATTGGTGCTAAAAATGGGGAAATGTCCAACTATTTCATGTTTACCTCATGAACTGACCCAAATGTCAAGAAGTATGTGTCTTAAAGTATGTTGTGGCCTGAAGAACTGCCACATCTAATGTCCCCTTCAAAAAACCAGGAAATATTGCAGGCAAAAGTAACTGCCTCCATACGATGGATGGAGATTTTTGATTAGATTCTTTATTTAATGAAAAATCAGAAGTTACAATTCTATTTTGTCTGTATAATGATTAAGCCACTTTTCTCATTTATAGATGTTTGCATTATCTGAAAAATGCATCAATTTAATTTCTGTAGTCAAAGTAACGGCTGTGCTTTCCTCTGGGGCGGGGTGGTGGTGGTGTTGTGATAAACCTTTCGTTACTAATTCAGAAGAGCAGATAATGTCCCTGCCGGCTGTAACTTTCTCTTTGGTTTTAATTTTTCTTCCCTGTTGTCAGGAGCCTTCCAGTGGCAAGAAAAGGATCTATTTCGGATTTGTTGTACATGGCGTGGTTAGAAATCCTGACCAAGAACCTTCCTCCTGTGTTACTGGTTAGAGGAAATCACAAAAATGTCTTAACATTTTACTCTTAAAGCCTGAAGGATGAGAAATGTTGTGCCTTGGTGTCTGAATAATTCTGAAACACATCTGGTTCTTTGTGTTTTAAAACCTCTTCTATGAATATACAAACCTCTGGAGACTAACCTAACCGATTCTACATAAGTTTTGTCTTTTAAAAAAAAGTTATTATTAATGGGAGGTTTTTTTCCCACTAGCTTAAAAAACATTGAAAGCCATGAGACTCCTAGAAAAACACTTTTCCGTTGTTGCTGGTAAATAAGAAATTAATAAAAGCTGGCTGACATGCTTACAGGATATGCTAATCTAGCTGTGAGCTCCAGGCAAACTTTATCTCGTTTCTGTAGAGTCTGCCGCATGGCCCTGGTGCAGGCCACAGGCCCACAGCGGGCTCTCCTTAAGTTCTTATTGACAGGCTGGTATGGTGAAGGCAGGTGGCTATCATAGGAAAAGGCCTGCTGGGTCAGTGGCCTGATTCTGCTTGGCCTCCTGAGAAGTTTGTGAAGATTTAAAAATCAATATCTGGAAACTCTAAGAAGGACTGAAGTCTACAAAGTGTTCAAAGCAGTTACCTAAATAAGGTTTATTTAATGCAGCGGATAATAAGCTTACCCTCTGATCACATGTAGCTTACACTTGCTGCTAGCTGGAGGTATATTAGGTAGGTGAGCAAGAGACCCATGTTCCACTTACATGAAATAGAGTCTTAACATACAAATATCTATTGTCTTCCTGGCTAAATATTTTGGTATTTCTACCTTAGTACTACTTTATTTTTTTCTCTCTCTAAGTGGTATGCATAGATGAAATAAAAAT

**Slc12a1-iCre #D1 (-12 bp)**

GAGCTGTGTATGTCCGTGGCTAACTCTGTACAGTGTACAGTGTGGGAACAGGTCAACAAAGAAAAAAGGCCACGAGCTTTTCCATTTTCATTTCTTATCGCATGGCCCTACCAATGTTAGGAAAATACCTACTGAAAATATTACTCATGAAATGAAGATTGGTGCTAAAAATGGGGAAATGTCCAACTATTTCATGTTTACCTCATGAACTGACCCAAATGTCAAGAAGTATGTGTCTTAAAGTATGTTGTGGCCTGAAGAACTGCCACATCTAATGTCCCCTTCAAAAAACCAGGAAATATTGCAGGCAAAAGTAACTGCCTCCATACGATGGATGGAGATTTTTGATTAGATTCTTTATTTAATGAAAAATCAGAAGTTACAATTCTATTTTGTCTGTATAATGATTAAGCCACTTTTCTCATTTATAGATGTTTGCATTATCTGAAAAATGCATCAATTTAATTTCTGTAGTCAAAGTAACGGCTGTGCTTTCCTCTGGGGCGGGGTGGTGGTGGTGTTGTGATAAACCTTTCGTTACTAATTCAGAAGAGCAGATAATGTCCCTGCCGGCTGTAACTTTCTCTTTGGTTTTAATTTTTCTTCCCTGTTGTCAGGAGCCTTCCAGTGGCAAGAAAAGGATCTATTTCGGATTTGTTGTACATGGCGTGGTTAGAAATCCTGACCAAGAACCTTCCTCCTGTGTTACTGGTTAGAGGAAATCACAAAAATGTCTTAACATTTTACTCTGAGGGCAGAGGAAGTCTTCTAACATGCGGTGACGTGGAGGAGAATCCCGGCCCTATGGTGCCCAAGAAGAAGAGGAAAGTCTCCAACCTGCTGACTGTGCACCAAAACCTGCCTGCCCTCCCTGTGGATGCCACCTCTGATGAAGTCAGGAAGAACCTGATGGACATGTTCAGGGACAGGCAGGCCTTCTCTGAACACACCTGGAAGATGCTCCTGTCTGTGTGCAGATCCTGGGCTGCCTGGTGCAAGCTGAACAACAGGAAATGGTTCCCTGCTGAACCTGAGGATGTGAGGGACTACCTCCTGTACCTGCAAGCCAGAGGCCTGGCTGTGAAGACCATCCAACAGCACCTGGGCCAGCTCAACATGCTGCACAGGAGATCTGGCCTGCCTCGCCCTTCTGACTCCAATGCTGTGTCCCTGGTGATGAGGAGAATCAGAAAGGAGAATGTGGATGCTGGGGAGAGAGCCAAGCAGGCCCTGGCCTTTGAACGCACTGACTTTGACCAAGTCAGATCCCTGATGGAGAACTCTGACAGATGCCAGGACATCAGGAACCTGGCCTTCCTGGGCATTGCCTACAACACCCTGCTGCGCATTGCCGAAATTGCCAGAATCAGAGTGAAGGACATCTCCCGCACCGATGGTGGGAGAATGCTGATCCACATTGGCAGGACCAAGACCCTGGTGTCCACAGCTGGTGTGGAGAAGGCCCTGTCCCTGGGGGTTACCAAGCTGGTGGAGAGATGGATCTCTGTGTCTGGTGTGGCTGATGACCCCAACAACTACCTGTTCTGCCGGGTCAGAAAGAATGGTGTGGCTGCCCCTTCTGCCACCTCCCAACTGTCCACCCGGGCCCTGGAAGGGATCTTTGAGGCCACCCACCGCCTGATCTATGGTGCCAAGGATGACTCTGGGCAGAGATACCTGGCCTGGTCTGGCCACTCTGCCAGAGTGGGTGCTGCCAGGGACATGGCCAGGGCTGGTGTGTCCATCCCTGAAATCATGCAGGCTGGTGGCTGGACCAATGTGAACATTGTGATGAACTACATCAGAAACCTGGACTCTGAGACTGGGGCCATGGTGAGGCTGCTCGAGGATGGGGACTAAAGTCTGGAGGATGAGAAATGTTGTGCCTTGGTGTCTGAATAATTCTGAAACACATCTGGTTCTTTGTGTTTTAAAACCTCTTCTATGAATATACAAACCTCTGGAGACTAACCTAACCGATTCTACATAAGTTTTGTCTTTTAAAAAAAAGTTATTATTAATGGGAGGTTTTTTTCCCACTAGCTTAAAAAACATTGAAAGCCATGAGACTCCTAGAAAAACACTTTTCCGTTGTTGCTGGTAAATAAGAAATTAATAAAAGCTGGCTGACATGCTTACAGGATATGCTAATCTAGCTGTGAGCTCCAGGCAAACTTTATCTCGTTTCTGTAGAGTCTGCCGCATGGCCCTGGTGCAGGCCACAGGCCCACAGCGGGCTCTCCTTAAGTTCTTATTGACAGGCTGGTATGGTGAAGGCAGGTGGCTATCATAGGAAAAGGCCTGCTGGGTCAGTGGCCTGATTCTGCTTGGCCTCCTGAGAAGTTTGTGAAGATTTAAAAATCAATATCTGGAAACTCTAAGAAGGACTGAAGTCTACAAAGTGTTCAAAGCAGTTACCTAAATAAGGTTTATTTAATGCAGCGGATAATAAGCTTACCCTCTGATCACATGTAGCTTACACTTGCTGCTAGCTGGAGGTATATTAGGTAGGTGAGCAAGAGACCCATGTTCCACTTACATGAAATAGAGTCTTAACATACAAATATCTATTGTCTTCCTGGCTAAATATTTTGGTATTTCTACCTTAGTACTACTTTATTTTTTTCTCTCTCTAAGTGGTATGCATAGATGAAATAAAAAT

**Plxnd1 WT**

NNN:TER

NNN:gRNA target

NNN:PAM

NNN:homology arm

NNN:P2A

NNN:iCre

NNN:ERT2

NNN:del

AAGAGGGAGGGAGGGAGGGAGGGCCAGAAGAGACCAACTGACCCTGGTCCAAAGTCTGACTGAATGGGGGTTGTTTAGCCATGTCTGAGGAGCCACCAAGGCCCTCTAGGTGGCAGTGGGGACAAGTCCCATCTTTCAGCCACCCAAACTGCCCGGGTCTCCCTGCCCAGGACTCACCCACCAACAAGCTTCTGTACGCGAAGGAGATCCCTGAGTACCGGAAGACCGTACAGCGCTATTATAAACAGATCCAAGACATGACGCCGCTCAGCGAGCAGGAAATGAACGCACACCTGGCCGAGGAGTCTCGGGTAGGTGGCCTTTGGGATCTAGGCCAGGAGATAGTAACGATATCCTTGGCTTTGAGGAGTGGACAAAACCAGCACCATTAGGATGCAGCAGGGGGACAGTAGGAATTTGGGGGTAGTCTCTGTTTCCCATCTTTCCTGGCATCTTCATTGCAATGTATTTTCTAAAGTCCAAGGATGAGGTGGAGTGTTCTGAAGATATGAGAGGTTAGAATCCTGGTTCCAGAAAGCCAGTGTGTGCCCAGCTCGCAGGGGTCTAAATGTATTCTATCAGCAGGCTGTGGGAGTGAACAGGCTGGGTCCTCATCTGTAGGGTTAAACCTCTCCTTTTGCTAGTTGGTAGTGGCACACACCTTTAATCCCAGCACTTGGGAGGTAGAGGCGGGTGGATTTCTGAGTCCGGGGCCAGCCTAGTCTACAGAGCAAGTTTCAGGACAGCCAGGGGGCTATGCAGAGAAACCCTGCCTCAAGCAAACAAGTAAAAATACCTCCCCTTTCTTCTTGTGCCACCTTTATAGAAATACCAGAATGAGTTCAACACAAACGTGGCCATGGCTGAGATTTATAAATATGCTAAGAGGTATCGACCACAGGTGAGTTCCCCCTCCCAAGATTTGCCCATTTTAGCCCATAACAACGCTTTAGATTTGTCGGGGGTGTTGGCACTTTGATGAGGTCTCTGGCGGGTGGCCTCCCAGGCTCTCAGGCACGGCACATCTGACTCACTGCAACTCCCCTCAGATCATGGCTGCCCTGGAGGCCAACCCCACAGCCCGCAGGACCCAGCTACAGCACAAGTTTGAACAGGTGGTGGCTCTGATGGAAAACAATATCTATGAGTGTTACAGCGAGGCCTGATGCAGAAGAGTGACCAGGAGCTTCGGCCAGGGAGACGGCGTGCAGGCCACTTGGCCTCCACTTGGTTTCTTCCCCACATCTCTCACTTGGGCTGGGAACTGACAGAGGAGCCTGCTGGGCTAGGAGTGGGGGACACTGGCCTCTTAGTGCCCGGCTGCCGAGCTCTTGGCCTTGTCCCCTGGGGCATCTCTGTCCCCTCCACCTGCCCAAGACCCAACTCTAGGATGAAGGCCTTGAATATCGATCGCTGCCAGTCCCTAATAAGACTTTCCCTGCCAACCAGGACAGCCTGGACCATGCCTGCCTGTTCACTGTTTCAGGCTGCTCAGCACACATTGGGAGAGGTGGCCATATCCCAGAACACTACCTCATCCACCTGGCAGAGGGAATTTCTGCTTCAGCCACCAAGCAGTTGTCTGTGTCCCTCATCCAGAGGGGGCCTTGGCCACCAACAGTTCCAAACCAGGTCAGCTGTTAGCCGTCTCATTGGCCAGTGGCAGCATGGGCAGTGCCCATTGCCCACAGAACGGTGGAGAGAGGGGGACAGGCTGGGGGGTTCCTGGCCCCAGGAAAGGGAGGAAGGCGAGGATGCAGGGCTGTAGCTGGACTACTCAGTCTTCCTGGAAGTGTTTCTAAAGAGCACCACTTTTTTTTGATTTTTAAGAAAAAAAAACTTTTATATATTAAAACAAAAACTTATGCACCAACTGTGAATAGCTGCCGCTTGTGCAGATCCCCAGGGGCTCCCGGTGACACACTGGAAATGACTGTTCCAGGGGACAGAAAATACTCATCTGTCCCCAGCACAGCCCCCACCCCACCCCCCATAGCTGCTGAGACTGGCTCACAGCCCAAGGGGGCTGGGCTGGAGGGGAAGGCTGGGACTCTCTGGAACATTCTTTATAATAAAAGCCTGCCGGGAAAACCTACTATGGGCTTTAGCCTTTGTCCTGAGATGGCCGAGGAGGGGAGATGGGGCTGTTTCTTCGTCCTGTCTGTGCAGAGCCTGACCTGACACCCTGCCTCGGACACGGCCTAAGGGAAGACCTCTCCCTTAGGCCTGCCAGGAGTTTGCGGC

**Plxnd1-ERT2-iCre-ERT2 #1 (-4 bp)**

aagagggagggagggagggagggccagaagagaccaactgaccctggtccaaagtctgactgaatgggggttgtttagccatgtctgaggagccaccaaggccctctaggtggcagtggggacaagtcccatctttcagccacccaaactgcccgggtctccctgcccaggactcacccaccaacaagcttctgtacgcgaaggagatccctgagtaccggaagaccgtacagcgctattataaacagatccaagacatgacgccgctcagcgagcaggaaatgaacgcacacctggccgaggagtctcgggtaggtggcctttgggatctaggccaggagatagtaacgatatccttggctttgaggagtggacaaaaccagcaccattaggatgcagcagggggacagtaggaatttgggggtagtctctgtttcccatctttcctggcatcttcattgcaatgtattttctaaagtccaaggatgaggtggagtgttctgaagatatgagaggttagaatcctggttccagaaagccagtgtgtgcccagctcgcaggggtctaaatgtattctatcagcaggctgtgggagtgaacaggctgggtcctcatctgtagggttaaacctctccttttgctagttggtagtggcacacacctttaatcccagcacttgggaggtagaggcgggtggatttctgagtccggggccagcctagtctacagagcaagtttcaggacagccagggggctatgcagagaaaccctgcctcaagcaaacaagtaaaaatacctcccctttcttcttgtgccacctttatagaaataccagaatgagttcaacacaaacgtggccatggctgagatttataaatatgctaagaggtatcgaccacaggtgagttccccctcccaagatttgcccattttagcccataacaacgctttagatttgtcgggggtgttggcactttgatgaggtctctggcgggtggcctcccaggctctcaggcacggcacatctgactcactgcaactcccctcagatcatggctgccctggaggccaaccccacagcccgcaggacccagctacagcacaagtttgaacaggtggtggctctgatggaaaacaatatctatgagtgttacagcgaggccggaagcggagctactaacttcagcctgctgaagcaggctggagacgtggaggagaaccctggacctatggctggagacatgagagctgccaacctttggccaagcccgctcatgatcaaacgctctaagaagaacagcctggccttgtccctgacggccgaccagatggtcagtgccttgttggatgctgagccccccatactctattccgagtatgatcctaccagacccttcagtgaagcttcgatgatgggcttactgaccaacctggcagacagggagctggttcacatgatcaactgggcgaagagggtgccaggctttgtggatttgaccctccatgatcaggtccaccttctagaatgtgcctggctagagatcctgatgattggtctcgtctggcgctccatggagcacccagtgaagctactgtttgctcctaacttgctcttggacaggaaccagggaaaatgtgtagagggcatggtggagatcttcgacatgctgctggctacatcatctcggttccgcatgatgaatctgcagggagaggagtttgtgtgcctcaaatctattattttgcttaattctggagtgtacacatttctgtccagcaccctgaagtctctggaagagaaggaccatatccaccgagtcctggacaagatcacagacactttgatccacctgatggccaaggcaggcctgaccctgcagcagcagcaccagcggctggcccagctcctcctcatcctctcccacatcaggcacatgagtaacaaaggcatggagcatctgtacagcatgaagtgcaagaacgtggtgcccctctatgacctgctgctggaggcggcggacgcccaccgcctacatgcgcccactagccgtggaggggcatccgtggaggagacggaccaaagccacttggccactgcgggctctacttcatcgcattccttgcaaaagtattacatcacgggggaggcagagggtttccctgccacagctgtcgacaacctgctgactgtgcaccaaaacctgcctgccctccctgtggatgccacctctgatgaagtcaggaagaacctgatggacatgttcagggacaggcaggccttctctgaacacacctggaagatgctcctgtctgtgtgcagatcctgggctgcctggtgcaagctgaacaacaggaaatggttccctgctgaacctgaggatgtgagggactacctcctgtacctgcaagccagaggcctggctgtgaagaccatccaacagcacctgggccagctcaacatgctgcacaggagatctggcctgcctcgcccttctgactccaatgctgtgtccctggtgatgaggagaatcagaaaggagaatgtggatgctggggagagagccaagcaggccctggcctttgaacgcactgactttgaccaagtcagatccctgatggagaactctgacagatgccaggacatcaggaacctggccttcctgggcattgcctacaacaccctgctgcgcattgccgaaattgccagaatcagagtgaaggacatctcccgcaccgatggtgggagaatgctgatccacattggcaggaccaagaccctggtgtccacagctggtgtggagaaggccctgtccctgggggttaccaagctggtggagagatggatctctgtgtctggtgtggctgatgaccccaacaactacctgttctgccgggtcagaaagaatggtgtggctgccccttctgccacctcccaactgtccacccgggccctggaagggatctttgaggccacccaccgcctgatctatggtgccaaggatgactctgggcagagatacctggcctggtctggccactctgccagagtgggtgctgccagggacatggccagggctggtgtgtccatccctgaaatcatgcaggctggtggctggaccaatgtgaacattgtgatgaactacatcagaaacctggactctgagactggggccatggtgaggctgctcgaggatggggacctcgagccatctgctggagacatgagagctgccaacctttggccaagcccgctcatgatcaaacgctctaagaagaacagcctggccttgtccctgacggccgaccagatggtcagtgccttgttggatgctgagccccccatactctattccgagtatgatcctaccagacccttcagtgaagcttcgatgatgggcttactgaccaacctggcagacagggagctggttcacatgatcaactgggcgaagagggtgccaggctttgtggatttgaccctccatgatcaggtccaccttctagaatgtgcctggctagagatcctgatgattggtctcgtctggcgctccatggagcacccagtgaagctactgtttgctcctaacttgctcttggacaggaaccagggaaaatgtgtagagggcatggtggagatcttcgacatgctgctggctacatcatctcggttccgcatgatgaatctgcagggagaggagtttgtgtgcctcaaatctattattttgcttaattctggagtgtacacatttctgtccagcaccctgaagtctctggaagagaaggaccatatccaccgagtcctggacaagatcacagacactttgatccacctgatggccaaggcaggcctgaccctgcagcagcagcaccagcggctggcccagctcctcctcatcctctcccacatcaggcacatgagtaacaaaggcatggagcatctgtacagcatgaagtgcaagaacgtggtgcccctctatgacctgctgctggaggcggcggacgcccaccgcctacatgcgcccactagccgtggaggggcatccgtggaggagacggaccaaagccacttggccactgcgggctctacttcatcgcattccttgcaaaagtattacatcacgggggaggcagagggtttccctgccacagcttgatgcagaagagtgaccaggagcttcggccagggagacggcgtgcaggccacttggcctccacttggtttcttccccacatctctcacttgggctgggaactgacagaggagcctgctgggctaggagtgggggacactggcctcttagtgcccggctgccgagctcttggccttgtcccctggggcatctctgtcccctccacctgcccaagacccaactctaggatgaaggccttgaatatcgatcgctgccagtccctaataagactttccctgccaaccaggacagcctggaccatgcctgcctgttcactgtttcaggctgctcagcacacattgggagaggtggccatatcccagaacactacctcatccacctggcagagggaatttctgcttcagccaccaagcagttgtctgtgtccctcatccagagggggccttggccaccaacagttccaaaccaggtcagctgttagccgtctcattggccagtggcagcatgggcagtgcccattgcccacagaacggtggagagagggggacaggctggggggttcctggccccaggaaagggaggaaggcgaggatgcagggctgtagctggactactcagtcttcctggaagtgtttctaaagagcaccacttttttttgatttttaagaaaaaaaaacttttatatattaaaacaaaaacttatgcaccaactgtgaatagctgccgcttgtgcagatccccaggggctcccggtgacacactggaaatgactgttccaggggacagaaaatactcatctgtccccagcacagcccccaccccaccccccatagctgctgagactggctcacagcccaagggggctgggctggaggggaaggctgggactctctggaacattctttataataaaagcctgccgggaaaacctactatgggctttagcctttgtcctgagatggccgaggaggggagatggggctgtttcttcgtcctgtctgtgcagagcctgacctgacaccctgcctcggacacggcctaagggaagacctctcccttaggcctgccaggagtttgcggc

**Plxnd1-ERT2-iCre-ERT2 #D1 (-19 bp)**

aagagggagggagggagggagggccagaagagaccaactgaccctggtccaaagtctgactgaatgggggttgtttagccatgtctgaggagccaccaaggccctctaggtggcagtggggacaagtcccatctttcagccacccaaactgcccgggtctccctgcccaggactcacccaccaacaagcttctgtacgcgaaggagatccctgagtaccggaagaccgtacagcgctattataaacagatccaagacatgacgccgctcagcgagcaggaaatgaacgcacacctggccgaggagtctcgggtaggtggcctttgggatctaggccaggagatagtaacgatatccttggctttgaggagtggacaaaaccagcaccattaggatgcagcagggggacagtaggaatttgggggtagtctctgtttcccatctttcctggcatcttcattgcaatgtattttctaaagtccaaggatgaggtggagtgttctgaagatatgagaggttagaatcctggttccagaaagccagtgtgtgcccagctcgcaggggtctaaatgtattctatcagcaggctgtgggagtgaacaggctgggtcctcatctgtagggttaaacctctccttttgctagttggtagtggcacacacctttaatcccagcacttgggaggtagaggcgggtggatttctgagtccggggccagcctagtctacagagcaagtttcaggacagccagggggctatgcagagaaaccctgcctcaagcaaacaagtaaaaatacctcccctttcttcttgtgccacctttatagaaataccagaatgagttcaacacaaacgtggccatggctgagatttataaatatgctaagaggtatcgaccacaggtgagttccccctcccaagatttgcccattttagcccataacaacgctttagatttgtcgggggtgttggcactttgatgaggtctctggcgggtggcctcccaggctctcaggcacggcacatctgactcactgcaactcccctcagatcatggctgccctggaggccaaccccacagcccgcaggacccagctacagcacaagtttgaacaggtggtggctctgatggaaaacaatatctatgagtgttacagcgaggccggaagcggagctactaacttcagcctgctgaagcaggctggagacgtggaggagaaccctggacctatggctggagacatgagagctgccaacctttggccaagcccgctcatgatcaaacgctctaagaagaacagcctggccttgtccctgacggccgaccagatggtcagtgccttgttggatgctgagccccccatactctattccgagtatgatcctaccagacccttcagtgaagcttcgatgatgggcttactgaccaacctggcagacagggagctggttcacatgatcaactgggcgaagagggtgccaggctttgtggatttgaccctccatgatcaggtccaccttctagaatgtgcctggctagagatcctgatgattggtctcgtctggcgctccatggagcacccagtgaagctactgtttgctcctaacttgctcttggacaggaaccagggaaaatgtgtagagggcatggtggagatcttcgacatgctgctggctacatcatctcggttccgcatgatgaatctgcagggagaggagtttgtgtgcctcaaatctattattttgcttaattctggagtgtacacatttctgtccagcaccctgaagtctctggaagagaaggaccatatccaccgagtcctggacaagatcacagacactttgatccacctgatggccaaggcaggcctgaccctgcagcagcagcaccagcggctggcccagctcctcctcatcctctcccacatcaggcacatgagtaacaaaggcatggagcatctgtacagcatgaagtgcaagaacgtggtgcccctctatgacctgctgctggaggcggcggacgcccaccgcctacatgcgcccactagccgtggaggggcatccgtggaggagacggaccaaagccacttggccactgcgggctctacttcatcgcattccttgcaaaagtattacatcacgggggaggcagagggtttccctgccacagctgtcgacaacctgctgactgtgcaccaaaacctgcctgccctccctgtggatgccacctctgatgaagtcaggaagaacctgatggacatgttcagggacaggcaggccttctctgaacacacctggaagatgctcctgtctgtgtgcagatcctgggctgcctggtgcaagctgaacaacaggaaatggttccctgctgaacctgaggatgtgagggactacctcctgtacctgcaagccagaggcctggctgtgaagaccatccaacagcacctgggccagctcaacatgctgcacaggagatctggcctgcctcgcccttctgactccaatgctgtgtccctggtgatgaggagaatcagaaaggagaatgtggatgctggggagagagccaagcaggccctggcctttgaacgcactgactttgaccaagtcagatccctgatggagaactctgacagatgccaggacatcaggaacctggccttcctgggcattgcctacaacaccctgctgcgcattgccgaaattgccagaatcagagtgaaggacatctcccgcaccgatggtgggagaatgctgatccacattggcaggaccaagaccctggtgtccacagctggtgtggagaaggccctgtccctgggggttaccaagctggtggagagatggatctctgtgtctggtgtggctgatgaccccaacaactacctgttctgccgggtcagaaagaatggtgtggctgccccttctgccacctcccaactgtccacccgggccctggaagggatctttgaggccacccaccgcctgatctatggtgccaaggatgactctgggcagagatacctggcctggtctggccactctgccagagtgggtgctgccagggacatggccagggctggtgtgtccatccctgaaatcatgcaggctggtggctggaccaatgtgaacattgtgatgaactacatcagaaacctggactctgagactggggccatggtgaggctgctcgaggatggggacctcgagccatctgctggagacatgagagctgccaacctttggccaagcccgctcatgatcaaacgctctaagaagaacagcctggccttgtccctgacggccgaccagatggtcagtgccttgttggatgctgagccccccatactctattccgagtatgatcctaccagacccttcagtgaagcttcgatgatgggcttactgaccaacctggcagacagggagctggttcacatgatcaactgggcgaagagggtgccaggctttgtggatttgaccctccatgatcaggtccaccttctagaatgtgcctggctagagatcctgatgattggtctcgtctggcgctccatggagcacccagtgaagctactgtttgctcctaacttgctcttggacaggaaccagggaaaatgtgtagagggcatggtggagatcttcgacatgctgctggctacatcatctcggttccgcatgatgaatctgcagggagaggagtttgtgtgcctcaaatctattattttgcttaattctggagtgtacacatttctgtccagcaccctgaagtctctggaagagaaggaccatatccaccgagtcctggacaagatcacagacactttgatccacctgatggccaaggcaggcctgaccctgcagcagcagcaccagcggctggcccagctcctcctcatcctctcccacatcaggcacatgagtaacaaaggcatggagcatctgtacagcatgaagtgcaagaacgtggtgcccctctatgacctgctgctggaggcggcggacgcccaccgcctacatgcgcccactagccgtggaggggcatccgtggaggagacggaccaaagccacttggccactgcgggctctacttcatcgcattccttgcaaaagtattacatcacgggggaggcagagggtttccctgccacagcttgatgcagaagagtgaccaggagcttcggccagggagacggcgtgcaggccacttggcctccacttggtttcttccccacatctctcacttgggctgggaactgacagaggagcctgctgggctaggagtgggggacactggcctcttagtgcccggctgccgagctcttggccttgtcccctggggcatctctgtcccctccacctgcccaagacccaactctaggatgaaggccttgaatatcgatcgctgccagtccctaataagactttccctgccaaccaggacagcctggaccatgcctgcctgttcactgtttcaggctgctcagcacacattgggagaggtggccatatcccagaacactacctcatccacctggcagagggaatttctgcttcagccaccaagcagttgtctgtgtccctcatccagagggggccttggccaccaacagttccaaaccaggtcagctgttagccgtctcattggccagtggcagcatgggcagtgcccattgcccacagaacggtggagagagggggacaggctggggggttcctggccccaggaaagggaggaaggcgaggatgcagggctgtagctggactactcagtcttcctggaagtgtttctaaagagcaccacttttttttgatttttaagaaaaaaaaacttttatatattaaaacaaaaacttatgcaccaactgtgaatagctgccgcttgtgcagatccccaggggctcccggtgacacactggaaatgactgttccaggggacagaaaatactcatctgtccccagcacagcccccaccccaccccccatagctgctgagactggctcacagcccaagggggctgggctggaggggaaggctgggactctctggaacattctttataataaaagcctgccgggaaaacctactatgggctttagcctttgtcctgagatggccgaggaggggagatggggctgtttcttcgtcctgtctgtgcagagcctgacctgacaccctgcctcggacacggcctaagggaagacctctcccttaggcctgccaggagtttgcggc

**Cdkn2d WT**

NNN:gRNA target

NNN:PAM

NNN:homology arm

NNN:E2A,T2A

NNN:tdTomato

NNN:DTR

NNN:plasmid backbone

NNN:ins

NNN:ND

AATTCGATGCCTGTGACCTCTGTGAGCACCGTGCAAGCGGTGTATACACCTAGTTCCTAAACCGTAAACGTCCCACTTCTTGGAGACGTGATTAAACCACGTATCAGTATAAGCCTCCTTTTCCTTTCAAAACCTTCAGCAACCAGGAAGCCTTTCTTTTCTTGGATCCCCCCCACCCCCCACCCCCACACACAGCCCATAAAATGTCAGGCTTAAAGTTTTTCATCTGCACCGGGCAGTGGTGGCACACGCCTTTAGTCCTAGCACTTGGGAGGCAGAGGCAGGCGGATTTCTGAGTTTGAGGCCAGCCTGGTCTACAAAGTGAGTTCCAGGACTGCCAGGGCTACACAGAGAAACCCTGCTTTGGGTGGGGGGGAGCTTTTCATTTGCTAAGGAGAAAATATTTTAATTAAAATAGGATATATGAAGCAGAATACGGAAATAACACAAATGTTTAGTGTAAAACATGGTTTAGACGATATTAGAATAGATCCTGTTTCGACTGGAACAAGTGTGCAACAGTCTCCTTCTGGCCTTTGGAAGTCAGTGTTAGATTCTAAGCTGCAAACAAGAAGAGGGCTCGAGGTGGGTCCTAATTTGATGGCCCCCATCCCTCCAAGTGCACAGGGCAGGTGTTCGAGAAAACCTGTTTTCAGTGAGTAGGTTTGCATAACGGAGCTGAGGAGGCGTGGCCAGGATAAAGAAGTGGTTAGAGAAGCTGCTAAGGAGAGCTGGAGTGAGGAACGCAGGGCACAGAGACCGTGGGAACAGTTGCAGAAGGGTGGGGGACTAGCGAGTGAAAGCCAGTCTTATGGGAGCCCTGAAGAGCTTAAAAGGAGACTCCAGATCCCATTAGTAGTTATAGAGGAGGGAAACGGAAAAGATCACTGCGGAGGGCCCTCAGTCTTCCAAAGACTGGCCTTCTGCTCCCGGCCTGGAAGGCACCGATGGAGGGGGCAAGGAGACAACACTCCTACCGCCGCTGCGGGCCGGGTAAGATAGATTTGCCCTTGGCTTTGAGTCCTGGTTCTTCCCCACACACACCCACACCCCACCCCCCTGCTGCCAAGAGAGAGCCAGTCCCACCGCCAAGCACAAGCGGCTATCGCGTGGTGGCTAACGGGTATCAGAGACTGGAGATGCTCAGAAGGCCACCTAGAGAGCCTAGCAAAGGGGGTTGGAGGGAGAGGAATGCTGCACACGCCGGGAGCAGTGCAGAAGCGGAGCACGGCTGGCTGGCACCTAGGACAGCTTTATATAATATTGTTTTCTCTCTTTTCTTTGCAGGGCCCTGGAACTTCGCGGCCAATCCCAAGAGCAGAGCTAAATCCGGCCTCAGCCCGCCTTTTTCTTCTTAGCTTCACTTCTAGCGATGCTAGCGTGTCTAGCATGTGGCTTTAAAAAATACATAATAATGCTTTTTTTGCAATCACGGGAGGGAGCAGAGGGAGGGAGCAGAAGGAGGGAGGGAGGGAGGGAGGGACCTGGACAGGAAAGGAATGGCATGAGAAACTGAGCGAAGGCGGCCGCGAAGGGAATAATGGCTGGATTGTTTAAAAAAATAAAATAAAGATACTTTTTAAAATGTCAACTGATTTTTAGCATAGAAATGCTTTCCTCTGTCCCCAAACTCTCACTGAAGCTTCCATTTCTATATCATTTTACAGAAGGTGTTTGGAAACTTCTGTTTCTTCTGAAACTACCCCTCTGTAGCACCGTTTATTGGTTTCTTGTGAGCTGTACTCTGGTTCCCCATCCCATCCTCTACCCGATCCTGTTGGCGTCAGTCACAACAGCCATTTAGCGAAATAAAATTCAAAAAGGGCTACGTGACTCTTCTAAAGTTTCCAGCAAATTGGGAGCTCCGAGATTCTTAGCTTAAAAACGTAGTTTGTATCCGCCCATTCCGGCAACATCCTATTAGGGCACAGTGCCAAAGATTAACGAATT

**Cdkn2d-tdT-DTR #9 (+1 bp)**

aattcgatgcctgtgacctctgtgagcaccgtgcaagcggtgtatacacctagttcctaaaccgtaaacgtcccacttcttggagacgtgattaaaccacgtatcagtataagcctccttttcctttcaaaaccttcagcaaccaggaagcctttcttttcttggatcccccccaccccccacccccacacacagcccataaaatgtcaggcttaaagtttttcatctgcaccgggcagtggtggcacacgcctttagtcctagcacttgggaggcagaggcaggcggatttctgagtttgaggccagcctggtctacaaagtgagttccaggactgccagggctacacagagaaaccctgctttgggtgggggggagcttttcatttgctaaggagaaaatattttaattaaaataggatatatgaagcagaatacggaaataacacaaatgtttagtgtaaaacatggtttagacgatattagaatagatcctgtttcgactggaacaagtgtgcaacagtctccttctggcctttggaagtcagtgttagattctaagctgcaaacaagaagagggctcgaggtgggtcctaatttgatggcccccatccctccaagtgcacagggcaggtgttcgagaaaacctgttttcagtgagtaggtttgcataacggagctgaggaggcgtggccaggataaagaagtggttagagaagctgctaaggagagctggagtgaggaacgcagggcacagagaccgtgggaacagttgcagaagggtgggggactagcgagtgaaagccagtcttatgggagccctgaagagcttaaaaggagactccagatcccattagtagttatagaggagggaaacggaaaagatcactgcggagggccctcagtcttccaaagactggccttctgctcccggcctggaaggcaccgatggagggggcaaggagacaacactcctaccgccgctgcgggccgggtaagatagatttgcccttggctttgagtcctggttcttccccacacacacccacaccccacccccctgctgccaagagagagccagtcccaccgccaagcacaagcggctatcCgcgtggtggctaacgggtatcagagactggagatgctcagaaggccacctagagagcctagcaaagggggttggagggagaggaatgctgcacacgccgggagcagtgcagaagcggagcacggctggctggcacctaggacagctttatataatattgttttctctcttttctttgcagggccctggaacttcgcggccaatcccaagagcagagcGGAAGCGGACAGTGTACTAATTATGCTCTCTTGAAATTGGCTGGAGATGTTGAGAGCAACCCTGGACCTATGGTGAGCAAGGGCGAGGAGGTCATCAAAGAGTTCATGCGCTTCAAGGTGCGCATGGAGGGCTCCATGAACGGCCACGAGTTCGAGATCGAGGGCGAGGGCGAGGGCCGCCCCTACGAGGGCACCCAGACCGCCAAGCTGAAGGTGACCAAGGGCGGCCCCCTGCCCTTCGCCTGGGACATCCTGTCCCCCCAGTTCATGTACGGCTCCAAGGCGTACGTGAAGCACCCCGCCGACATCCCCGATTACAAGAAGCTGTCCTTCCCCGAGGGCTTCAAGTGGGAGCGCGTGATGAACTTCGAGGACGGCGGTCTGGTGACCGTGACCCAGGACTCCTCCCTGCAGGACGGCACGCTGATCTACAAGGTGAAGATGCGCGGCACCAACTTCCCCCCCGACGGCCCCGTAATGCAGAAGAAGACCATGGGCTGGGAGGCCTCCACCGAGCGCCTGTACCCCCGCGACGGCGTGCTGAAGGGCGAGATCCACCAGGCCCTGAAGCTGAAGGACGGCGGCCACTACCTGGTGGAGTTCAAGACCATCTACATGGCCAAGAAGCCCGTGCAACTGCCCGGCTACTACTACGTGGACACCAAGCTGGACATCACCTCCCACAACGAGGACTACACCATCGTGGAACAGTACGAGCGCTCCGAGGGCCGCCACCACCTGTTCCTGGGGCATGGCACCGGCAGCACCGGCAGCGGCAGCTCCGGCACCGCCTCCTCCGAGGACAACAACATGGCCGTCATCAAAGAGTTCATGCGCTTCAAGGTGCGCATGGAGGGCTCCATGAACGGCCACGAGTTCGAGATCGAGGGCGAGGGCGAGGGCCGCCCCTACGAGGGCACCCAGACCGCCAAGCTGAAGGTGACCAAGGGCGGCCCCCTGCCCTTCGCCTGGGACATCCTGTCCCCCCAGTTCATGTACGGCTCCAAGGCGTACGTGAAGCACCCCGCCGACATCCCCGATTACAAGAAGCTGTCCTTCCCCGAGGGCTTCAAGTGGGAGCGCGTGATGAACTTCGAGGACGGCGGTCTGGTGACCGTGACCCAGGACTCCTCCCTGCAGGACGGCACGCTGATCTACAAGGTGAAGATGCGCGGCACCAACTTCCCCCCCGACGGCCCCGTAATGCAGAAGAAGACCATGGGCTGGGAGGCCTCCACCGAGCGCCTGTACCCCCGCGACGGCGTGCTGAAGGGCGAGATCCACCAGGCCCTGAAGCTGAAGGACGGCGGCCGCTACCTGGTGGAGTTCAAGACCATCTACATGGCCAAGAAGCCCGTGCAACTGCCCGGCTACTACTACGTGGACACCAAGCTGGACATCACCTCCCACAACGAGGACTACACCATCGTGGAACAGTACGAGCGCTCCGAGGGCCGCCACCACCTGTTCCTGTACGGCATGGACGAGCTGTACAAGGGAAGCGGAGAGGGCAGAGGAAGTCTGCTAACATGCGGTGACGTCGAGGAGAATCCTGGACCTATGAAGCTGCTGCCGTCGGTGGTGCTGAAGCTCTTTCTGGCTGCAGTTCTCTCGGCACTGGTGACTGGCGAGAGCCTGGAGCGGCTTCGGAGAGGGCTAGCTGCTGGAACCAGCAACCCGGACCCTCCCACTGTATCCACGGACCAGCTGCTACCCCTAGGAGGCGGCCGGGACCGGAAAGTCCGTGACTTGCAAGAGGCAGATCTGGACCTTTTGAGAGTCACTTTATCCTCCAAGCCACAAGCACTGGCCACACCAAACAAGGAGGAGCACGGGAAAAGAAAGAAGAAAGGCAAGGGGCTAGGGAAGAAGAGGGACCCATGTCTTCGGAAATACAAGGACTTCTGCGTGCATGGAGAATGCAAATATGTGAAGGAGCTCCGGGCTCCCTCCTGCATCTGCCACCCGGGTTACCATGGAGAGAGGTGTCATGGGCTGAGCGTGCCAGTGGAAAATCGCTTATATACCTATGACCACACAACCATCCTGGCCGTGGTGGCTGTGGTGCTGTCATCTGTCTGTCTGCTGGTCATCGTGGGGCTTCTCATGTTTAGGTACCATAGGAGAGGAGGTTATGATGTGGAAAATGAAGAGAAAGTGAAGTTGGGCATGACTAATTCCCACTAAGGATCCCGcctcagcccgcctttttcttcttagcttcacttctagcgatgctagcgtgtctagcatgtggctttaaaaaatacataataatgctttttttgcaatcacgggagggagcagagggagggagcagaaggagggagggagggagggagggacctggacaggaaaggaatggcatgagaaactgagcgaaggcggccgcgaagggaataatggctggattgtttaaaaaaataaaataaagatactttttaaaatgtcaactgatttttagcatagaaatgctttcctctgtccccaaactctcactgaagcttccatttctatatcattttacagaaggtgtttggaaacttctgtttcttctgaaactacccctctgtagcaccgtttattggtttcttgtgagctgtactctggttccccatcccatcctctacccgatcctgttggcgtcagtcacaacagccatttagcgaaataaaattcaaaaagggctacgtgactcttctaaagtttccagcaaattgggagctccgagattcttagcttaaaaacgtagtttgtatccgcccattccggcaacatcctattagggcacagtgccaaagattaacgaattTGACTAGTAGATCCTCTAGAGTCGACCTGCAGGCATGCAAGCTTTCCCCTATAGTGTCACCTAAATAGCTTGGCGTAATCATGGTCATAGCTGTTTCCTGTGTGAAATTGTTATCCGCTCACAATTCCACACAACATACGAGCCGGAAGCATAAAGTGTAAAGCCTGGGGTGCCTAATGAGTGAGCTAACTCACATTAATTGCGTTGCGCTCACTGCCCGCTTTCCAGTCGGGAAACCTGTCGTGCCAGCTGCATTAATGAATCGGCCAACGCGCGGGGAGAGGCGGTTTGCGTATTGGGCGCTCTTCCGCTTCCTCGCTCACTGACTCGCTGCGCTCGGTCGTTCGGCTGCGGCGAGCGGTATCAGCTCACTCAAAGGCGGTAATACGGTTATCCACAGAATCAGGGGATAACGCAGGAAAGAACATGTGAGCAAAAGGCCAGCAAAAGGCCAGGAACCGTAAAAAGGCCGCGTTGCTGGCGTTTTTCCATAGGCTCCGCCCCCCTGACGAGCATCACAAAAATCGACGCTCAAGTCAGAGGTGGCGAAACCCGACAGGACTATAAAGATACCAGGCGTTTCCCCCTGGAAGCTCCCTCGTGCGCTCTCCTGTTCCGACCCTGCCGCTTACCGGATACCTGTCCGCCTTTCTCCCTTCGGGAAGCGTGGCGCTTTCTCATAGCTCACGCTGTAGGTATCTCAGTTCGGTGTAGGTCGTTCGCTCCAAGCTGGGCTGTGTGCACGAACCCCCCGTTCAGCCCGACCGCTGCGCCTTATCCGGTAACTATCGTCTTGAGTCCAACCCGGTAAGACACGACTTATCGCCACTGGCAGCAGCCACTGGTAACAGGATTAGCAGAGCGAGGTATGTAGGCGGTGCTACAGAGTTCTTGAAGTGGTGGCCTAACTACGGCTACACTAGAAGAACAGTATTTGGTATCTGCGCTCTGCTGAAGCCAGTTACCTTCGGAAAAAGAGTTGGTAGCTCTTGATCCGGCAAACAAACCACCGCTGGTAGCGGTGGTTTTTTTGTTTGCAAGCAGCAGATTACGCGCAGAAAAAAAGGATCTCAAGAAGATCCTTTGATCTTTTCTACGGGGTCTGACGCTCAGTGGAACGAAAACTCACGTTAAGGGATTTTGGTCATGAGATTATCAAAAAGGATCTTCACCTAGATCCTTTTAAATTAAAAATGAAGTTTTAAATCAATCTAAAGTATATATGAGTAAACTTGGTCTGACAGTTACCAATGCTTAATCAGTGAGGCACCTATCTCAGCGATCTGTCTATTTCGTTCATCCATAGTTGCCTGACTCCCCGTCGTGTAGATAACTACGATACGGGAGGGCTTACCATCTGGCCCCAGTGCTGCAATGATACCGCGAGACCCACGCTCACCGGCTCCAGATTTATCAGCAATAAACCAGCCAGCCGGAAGGGCCGAGCGCAGAAGTGGTCCTGCAACTTTATCCGCCTCCATCCAGTCTATTAATTGTTGCCGGGAAGCTAGAGTAAGTAGTTCGCCAGTTAATAGTTTGCGCAACGTTGTTGCCATTGCTACAGGCATCGTGGTGTCACGCTCGTCGTTTGGTATGGCTTCATTCAGCTCCGGTTCCCAACGATCAAGGCGAGTTACATGATCCCCCATGTTGTGCAAAAAAGCGGTTAGCTCCTTCGGTCCTCCGATCGTTGTCAGAAGTAAGTTGGCCGCAGTGTTATCACTCATGGTTATGGCAGCACTGCATAATTCTCTTACTGTCATGCCATCCGTAAGATGCTTTTCTGTGACTGGTGAGTACTCAACCAAGTCATTCTGAGAATAGTGTATGCGGCGACCGAGTTGCTCTTGCCCGGCGTCAATACGGGATAATACCGCGCCACATAGCAGAACTTTAAAAGTGCTCATCATTGGAAAACGTTCTTCGGGGCGAAAACTCTCAAGGATCTTACCGCTGTTGAGATCCAGTTCGATGTAACCCACTCGTGCACCCAACTGATCTTCAGCATCTTTTACTTTCACCAGCGTTTCTGGGTGAGCAAAAACAGGAAGGCAAAATGCCGCAAAAAAGGGAATAAGGGCGACACGGAAATGTTGAATACTCATACTCTTCCTTTTTCAATATTATTGAAGCATTTATCAGGGTTATTGTCTCATGAGCGGATACATATTTGAATGTATTTAGAAAAATAAACAAATAGGGGTTCCGCGCACATTTCCCCGAAAAGTGCCACCTGACGTCTAAGAAACCATTATTATCATGACATTAACCTATAAAAATAGGCGTATCACGAGGCCCTTTCGTCTCGCGCGTTTCGGTGATGACGGTGAAAACCTCTGACACATGCAGCTCCCGGAGACGGTCACAGCTTGTCTGTAAGCGGATGCCGGGAGCAGACAAGCCCGTCAGGGCGCGTCAGCGGGTGTTGGCGGGTGTCGGGGCTGGCTTAACTATGCGGCATCAGAGCAGATTGTACTGAGAGTGCACTATATGCGGTGTGAAATACCGCACAGATGCGTAAGGAGAAAATACCGCATCAGGGCCATTCGCCATTCAGGCTGCGCAACTGTTGGGAAGGGCGATCGGTGCGGGCCTCTTCGCTATTACGCCAGCTGGCGAAAGGGGGATGTGCTGCAAGGCGATTAAGTTGGGTAACGCCAGGGTTTTCCCAGTCACGACGTTGTAAAACGACGGCCAGTGAATTcgatgcctgtgacctctgtgagcaccgtgcaagcggtgtatacacctagttcctaaaccgtaaacgtcccacttcttggagacgtgattaaaccacgtatcagtataagcctccttttcctttcaaaaccttcagcaaccaggaagcctttcttttcttggatcccccccaccccccacccccacacacagcccataaaatgtcaggcttaaagtttttcatctgcaccgggcagtggtggcacacgcctttagtcctagcacttgggaggcagaggcaggcggatttctgagtttgaggccagcctggtctacaaagtgagttccaggactgccagggctacacagagaaaccctgctttgggtgggggggaNNNNNNNNNNctctcttttctttgcagGGCCCTGGNNNNNNNNNNgcctcagcccgcctttttcttcttagcttcacttctagcgatgctagcgtgtctagcatgtggctttaaaaaatacataataatgctttttttgcaatcacgggagggagcagagggagggagcagaaggagggagggagggagggagggacctggacaggaaaggaatggcatgagaaactgagcgaaggcggccgcgaagggaataatggctggattgtttaaaaaaataaaataaagatactttttaaaatgtcaactgatttttagcatagaaatgctttcctctgtccccaaactctcactgaagcttccatttctatatcattttacagaaggtgtttggaaacttctgtttcttctgaaactacccctctgtagcaccgtttattggtttcttgtgagctgtactctggttccccatcccatcctctacccgatcctgttggcgtcagtcacaacagccatttagcgaaataaaattcaaaaagggctacgtgactcttctaaagtttccagcaaattgggagctccgagattcttagcttaaaaacgtagtttgtatccgcccattccggcaacatcctattagggcacagtgccaaagattaacgaattctcttgcaaaacaaacgacaggacag

**Rats**

**Pva-2A Cre WT**

nnn:intron

NNN:exon

NNN:TER

NNN:gRNA target

NNN:PAM

NNN:homology arm

NNN:T2A

NNN:Cre

NNN:bGH polyA

NNN:del

ctgtggtcaatttgaatttttaaagcattatttatatttgcacaatgtgacaggagcaggaagagtccctttgcccttctcccagcctccctggggttaacatttatgtaacctcggtagagggtgacatatgaggagtgacgtacaccgatgtcctatacacagactgggtagactgcctaaaccgctcactgatctcacaacggtgcatttccagaggtgacaggaattggcgggccagaacctcagggggttccagagactaggcttggtgataaacaatatggtggcagctgaagcccaagggggtattgtaagcgaagggtttaagatgactgctgcacggcagatgtgcgggtgattgccggcgataataatagtgtgtcctcttttcttttgcagAATTCTCCACTCTGGTGGCCGAAAGCTAAGTGGCGCTGACTGCTTGGGTCTCCCACCTCTCCACCCCCCATGCCCCATCTCAGCCCTTCTCGCGGCCCTCCTGGGTTTCTGTTCAGTTTGTTTATGTTATTTTTTACTCCCCCATCCTTTATGGCCCTCGAATGACACCACTCTTCTGGAAAATGCTGGAGAAACAATAAAGGCTGTACCCATCGGACACCacctgtagggaggacccaggcctggtagggtgttggtttggcaagtttttccggacagcagtgggggtatagtagaaaaagtgagagagagcgaaggaccacgccctgatatttcctgcctgcttggtaccgagtggtcacgtg

**Pva-2A Cre #2 (-1 bp)**

TATACCTGGGTTAAGGGAGGTCACAAATCTCCCCAGGAAAGAAGGGCTCTGAGAATGAGTGTCACCATGTTAAAGTTAAACTCTAGGGCCTCAGTTTCCCCATCAGTAAAATGCAGTCAGAGTGTTGTCTGTCTGTGGTAATTCTAAGAATTCCATGAGGTAACATTCCCTGGATCCCTCCCACACAGACGTTTGGTGAATGCCTTGCAGATTGTCAATGGGAACAACACAGACAGTGTGAATGAGAATTCTAACAACAGGATAAAGACAGGTCAAGAGAACCCAGCCCCCCTCCAGTCCTGCATGCTCTGGGGACTCTTGGCTGAGAAGACCGGTCCATTGACCCCTGCCCAGGGGATATTCTTTGAATGTTTGAGTCCCCATTAGTGCTTCTCCCCATTAAGTCCAGGATCCTTTTGAGATTCCAGGAGTGTCTTCACATGCATGGATTACCACATCCAGGGACACTGCTTTGAGGACAGGAGGGTGTGGTGTCTGTTTCCCAGGTTCCAGGCTTTGTCCCCATCACAGAGCCAGGGCCGGGACAGCTGCATGGTACGGCTGATCTTGCTGTGGTCAATTTGAATTTTTAAAGCATTATTTATATTTGCACAATGTGACAGGAGCAGGAAGAGTCCCTTTGCCCTTCTCCCAGCCTCCCTGGGGTTAACATTTATGTAACCTCGGTAGAGGGTGACATATGAGGAGTGACGTACACCGATGTCCTATACACAGACTGGGTAGACTGCCTAAACCGCTCACTGATCTCACAACGGTGCATTTCCAGAGGTGACAGGAATTGGCGGGCCAGAACCTCAGGGGGTTCCAGAGACTAGGCTTGGTGATAAACAATATGGTGGCAGCTGAAGCCCAAGGGGGTATTGTAAGCGAAGGGTTTAAGATGACTGCTGCACGGCAGATGTGCGGGTGATTGCCGGCGATAATAATAGTGTGTCCTCTTTTCTTTTGCAGAATTCTCCACTCTGGTGGCCGAAAGCGGAAGCGGAGAGGGCAGAGGAAGTCTTCTAACATGCGGTGACGTGGAGGAGAATCCCGGCCCTATGCCCAAGAAGAAGAGGAAGGTGTCCAATTTACTGACCGTACACCAAAATTTGCCTGCATTACCGGTCGATGCAACGAGTGATGAGGTTCGCAAGAACCTGATGGACATGTTCAGGGATCGCCAGGCGTTTTCTGAGCATACCTGGAAAATGCTTCTGTCCGTTTGCCGGTCGTGGGCGGCATGGTGCAAGTTGAATAACCGGAAATGGTTTCCCGCAGAACCTGAAGATGTTCGCGATTATCTTCTATATCTTCAGGCGCGCGGTCTGGCAGTAAAAACTATCCAGCAACATTTGGGCCAGCTAAACATGCTTCATCGTCGGTCCGGGCTGCCACGACCAAGTGACAGCAATGCTGTTTCACTGGTTATGCGGCGGATCCGAAAAGAAAACGTTGATGCCGGTGAACGTGCAAAACAGGCTCTAGCGTTCGAACGCACTGATTTCGACCAGGTTCGTTCACTCATGGAAAATAGCGATCGCTGCCAGGATATACGTAATCTGGCATTTCTGGGGATTGCTTATAACACCCTGTTACGTATAGCCGAAATTGCCAGGATCAGGGTTAAAGATATCTCACGTACTGACGGTGGGAGAATGTTAATCCATATTGGCAGAACGAAAACGCTGGTTAGCACCGCAGGTGTAGAGAAGGCACTTAGCCTGGGGGTAACTAAACTGGTCGAGCGATGGATTTCCGTCTCTGGTGTAGCTGATGATCCGAATAACTACCTGTTTTGCCGGGTCAGAAAAAATGGTGTTGCCGCGCCATCTGCCACCAGCCAGCTATCAACTCGCGCCCTGGAAGGGATTTTTGAAGCAACTCATCGATTGATTTACGGCGCTAAGGATGACTCTGGTCAGAGATACCTGGCCTGGTCTGGACACAGTGCCCGTGTCGGAGCCGCGCGAGATATGGCCCGCGCTGGAGTTTCAATACCGGAGATCATGCAAGCTGGTGGCTGGACCAATGTAAATATTGTCATGAACTATATCCGTAACCTGGATAGTGAAACAGGGGCAATGGTGCGCCTGCTGGAAGATGGCGACTAAACTAGTCTGTGCCTTCTAGTTGCCAGCCATCTGTTGTTTGCCCCTCCCCCGTGCCTTCCTTGACCCTGGAAGGTGCCACTCCCACTGTCCTTTCCTAATAAAATGAGGAAATTGCATCGCATTGTCTGAGTAGGTGTCATTCTATTCTGGGGGGTGGGGTGGGGCAGGACAGCAAGGGGGAGGATTGGGAAGACAATAGCAGGCATGCTGGGGATGCGGTGGGCTCTATGGGCGGCCGCGCTGACTGCTTGGGTCTCCCACCTCTCCACCCCCCATGCCCCATCTCAGCCCTTCTCGCGGCCCTCCTGGGTTTCTGTTCAGTTTGTTTATGTTATTTTTTACTCCCCCATCCTTTATGGCCCTCGAATGACACCACTCTTCTGGAAAATGCTGGAGAAACAATAAAGGCTGTACCCATCGGACACCACCTGTAGGGAGGACCCAGGCCTGGTAGGGTGTTGGTTTGGCAAGTTTTTCCGGACAGCAGTGGGGGTATAGTAGAAAAAGTGAGAGAGAGCGAAGGACCACGCCCTGATATTTCCTGCCTGCTTGGTACCGAGTGGTCACGTGGGCCACCTTGTTCAGTCTTTGTGCCTTTCCTACAAGGGGATGGGATGGCGCAGGGGATTTTAAAGATGCAGAAACTGCCTTTTAAAGAGCAGAACGGAAGGGGCTGAGTCCACAGGTGATTACTTTATGTCCCTGAGGAATAACTAGGTCGAAGGACTCAAATGACACTCTATCAATTGCTTTTGACTTTGCTGTGATAAAATTCCTGATAAGAGAAACTTAAGGAAAGAGCGGTTTCTTTTGGCTCTCTCAAAGGGTGTGGTCTATCATGGTAGGGAAGCCCCCAAGGCAGGCTGTGCAGTAGCAGGCCACACTGAATCACACTGGATCCACGGGTCAAAAAGGGAAGAGGCCAAGACCCGTGCTCAGCTGGCTTCTTCTTTTTCTTCAGCCTGGGACTCCAGACCATTGGGTGATGCTGCCCACATTTGAAGTTGGTCTTCCTACCTCAGGCTAAGCTACAAACTCCCTCATGGGTGTGCTCATGGGTGTGCCTGTAGGTTTATTTCCTGGACGATTCTAGGCTCCCTTCAAGTTGGCAAGATGAACCGTCGTCATAAATACCCACAAGAGTGAGCCCTTCCTTGCTTGCCACCGAGCACTGCCTCTCTTCCTGCCAAACTAGGCTCAGGCTTTCCTCCCAGTGTAAGCAGGACTACCAGGAAGTTCAAAGCATCATACGGCAAGACCAGCAGATGTGTGCTCCTCCTTCCCCACCACCGACCCAACCACCCCATCCCACACCCCCCACTCCCGTCCAAACTCAGCTGCACTCTTGGCCTTGCTAAGGTGTCAGGAGCATGTGTTTGCAGAAACCTTTGCAGGATGCCATTTGCAGCAGCCCTGCAAGGAATGCACTGTCACAACACCCCTGGGAATCCACAGAGCCTCAGACTGGAGGACATTGTTTCTCTAGGGTCACACTGACAGAAGCAGTAGAACCGGGACCCCGAGATTCCTGTGCCCCTCTACAAATGTGTCCCGATGGCCAGAAGGGCCCTGAGCGGGCCCCTGATGGGTCATACATCCTCAGAAATGAGGGTCAGTCACTCAGCTTAATCCTGAGGGAAAGGAGGAAAGAACCTCTTTGGAAATCTGAAGCCAAGGTAGACGGTAAGCCTGCAGA

**Th-2A Cre WT**

nnn:intron

NNN:exon

NNN:TER

NNN:gRNA target

NNN:PAM

NNN:homology arm

NNN:T2A

NNN:Cre

NNN:bGH polyA

NNN:del

NNN:ins

ggctggagtgggtggggacagataaggaagaacaagagacgaagtggaagctaagagctgccatgagggcaaaggttatagaaggggtaggcagtacaaattcaagggacacagttatacagggtctgagaaaggaagaggagactggaaagttggtccttatcacttctcctggcctgctgtcctggcatgtccacttcagtttagggactctctagggcacacagtcaaggagagcctcctgagggaagaagtccagatgagaaatgaagtgctttgcagaagtctgcccacaagattttctggggctttagtctcctgaatgtcaggaatgggctttaggggtttggcaaactcagaaaagccatttccccattctcctggaggttgcagattttatcccgagctacctgactctagcctgctttctcacccttacatccagtctcctgtgattgaggaatcataccggcttccctccacagGAACTATGCCTCTCGTATCCAGCGCCCATTCTCTGTGAAGTTTGACCCGTACACACTGGCCATTGACGTACTGGACAGCCCTCACACCATCCAGCGCTCCTTGGAGGGGGTCCAGGATGAGCTGCACACCCTGGCCCACGCACTGAGTGCCATTAGCTAAATGCATAGGGTACCACCCACAGGTGCCAGGGGCCTTTCCCAAAGTCTCCATCCCCTTCTCCAACCTTTCCTGGCCCAGAGGCTTTCCCATGTGTGTGGCTGGGCCCTTTGATGGGCTCCTCTTGGACCCCCATCCTCCCAACACTGCTTCTCAACCATGTCTTACTACTGCATGCACTCCAGGGTGGTCCTGCATTCCTCCTGCCCTCCATGCTCTATACTACCCTGATTATTCTCTCAATAAAGGAAGGAAAGATCTccagggctgcctctggtcactgcgtcaccacaaagatagttcctagcattcatatctgctctggcttcacttggaggtttagacggtttcttctcccccacctctttcttccaattcagcctcctggacacttcagaccagacagaaggtactggtgttatctccctgccaactatgagctctgaaacagtactggggggtgttgaacaaggccccagatgagctgccccagaagcatgggctaacaacacaaaggtggagaccagggtaaggagtg

**Th-2A Cre #3 (-548 bp)**

ACGAAGTGGAAGCTAAGAGCTGCCATGAGGGCAAAGGTTATAGAAGGGGTAGGCAGTACAAATTCAAGGGACACAGTTATACAGGGTCTGAGAAAGGAAGAGGAGACTGGAAAGTTGGTCCTTATCACTTCTCCTGGCCTGCTGTCCTGGCATGTCCACTTCAGTTTAGGGACTCTCTAGGGCACACAGTCAAGGAGAGCCTCCTGAGGGAAGAAGTCCAGATGAGAAATGAAGTGCTTTGCAGAAGTCTGCCCACAAGATTTTCTGGGGCTTTAGTCTCCTGAATGTCAGGAATGGGCTTTAGGGGTTTGGCAAACTCAGAAAAGCCATTTCCCCATTCTCCTGGAGGTTGCAGATTTTATCCCGAGCTACCTGACTCTAGCCTGCTTTCTCACCCTTACATCCAGTCTCCTGTGATTGAGGAATCATACCGGCTTCCCTCCACAGGAACTATGCCTCTCGTATCCAGCGCCCATTCTCTGTGAAGTTTGACCCGTACACACTGGCCATTGACGTACTGGACAGCCCTCACACCATCCAGCGCTCCTTGGAGGGGGTCCAGGATGAGCTGCACACCCTGGCCCACGCACTGAGTGCAATTAGCGGAAGCGGAGAGGGCAGAGGAAGTCTGCTAACATGCGGTGACGTGGAGGAGAATCCCGGCCCTATGCCCAAGAAGAAGAGGAAGGTGTCCAATTTACTGACCGTACACCAAAATTTGCCTGCATTACCGGTCGATGCAACGAGTGATGAGGTTCGCAAGAACCTGATGGACATGTTCAGGGATCGCCAGGCGTTTTCTGAGCATACCTGGAAAATGCTTCTGTCCGTTTGCCGGTCGTGGGCGGCATGGTGCAAGTTGAATAACCGGAAATGGTTTCCCGCAGAACCTGAAGATGTTCGCGATTATCTTCTATATCTTCAGGCGCGCGGTCTGGCAGTAAAAACTATCCAGCAACATTTGGGCCAGCTAAACATGCTTCATCGTCGGTCCGGGCTGCCACGACCAAGTGACAGCAATGCTGTTTCACTGGTTATGCGGCGGATCCGAAAAGAAAACGTTGATGCCGGTGAACGTGCAAAACAGGCTCTAGCGTTCGAACGCACTGATTTCGACCAGGTTCGTTCACTCATGGAAAATAGCGATCGCTGCCAGGATATACGTAATCTGGCATTTCTGGGGATTGCTTATAACACCCTGTTACGTATAGCCGAAATTGCCAGGATCAGGGTTAAAGATATCTCACGTACTGACGGTGGGAGAATGTTAATCCATATTGGCAGAACGAAAACGCTGGTTAGCACCGCAGGTGTAGAGAAGGCACTTAGCCTGGGGGTAACTAAACTGGTCGAGCGATGGATTTCCGTCTCTGGTGTAGCTGATGATCCGAATAACTACCTGTTTTGCCGGGTCAGAAAAAATGGTGTTGCCGCGCCATCTGCCACCAGCCAGCTATCAACTCGCGCCCTGGAAGGGATTTTTGAAGCAACTCATCGATTGATTTACGGCGCTAAGGATGACTCTGGTCAGAGATACCTGGCCTGGTCTGGACACAGTGCCCGTGTCGGAGCCGCGCGAGATATGGCCCGCGCTGGAGTTTCAATACCGGAGATCATGTGCCTCAGTAGAGCCAGGATCAAGCTGGTGGCTGGACCAATGTAAATATTGTCATGAACTATATCCGTAACCTGGATAGTGAAACAGGGGCAATGGTGCGCCTGCTGGAAGATGGCGACTAAACTAGTCTGTGCCTTCTAGTTGCCAGCCATCTGTTGTTTGCCCCTCCCCCGTGCCTTCCTTGACCCTGGAAGGTGCCACTCCCACTGTCCTTTCCTAATAAAATGAGGAAATTGCATCGCATTGTCTGAGTAGGTGTCATTCTATTCTGGGGGGTGGGGTGGGGCAGGACAGCAAGGGGGAGGATTGGGAAGACAATAGCAGGCATGCTGGGGATGCGGTGGGCTCTATGGGCGGCCGCTAGTGCATAGGGTACCACCCACAGGTGCCAGGGGCCTTTCCCAAAGTCTCCATCCCCTTCTCCAACCTTTCCTGGCCCAGAGGCTTTCCCATGTGTGTGGCTGGGCCCTTTGATGGGCTCCTCTTGGACCCCCATCCTCCCAACACTGCTTCTCAACCATGTCTTACTACTGCATGCACTCCAGGGTGGTCCTGCATTCCTCCTGCCCTCCATGCTCTATACTACCCTGATTATTCTCTCAATAAAGGAAGGAAAGATCTCCAGGGCTGCCTCTGGTCACTGCGTCACCACAAAGATAGTTCCTAGCATTCATATCTGCTCTGGCTTCACTTGGAGGTTTAGACGGTTTCTTCTCCCCCACCTCTTTCTTCCAATTCAGCCTCCTGGACACTTCAGACCAGACAGAAGGTACTGGTGTTATCTCCCTGCCAACTATGAGCTCTGAAACAGTACTGGGGGGTGTTGAACAAGGCCCCAGATGAGCTGCCCCAGAAGCATGGGCTAACAACACAAAGGTGGAGACCAGGGTAAGGAGTGACAGTCACAGCTGTGCTCACCAGGGAACAGCCTCTGACAAGGCTTGTAGACCAAGCCCTCTCCCTGTGCTCAGGAGTCTTAGGCACCTTGCAAGTAGGCAGAGGATTGAAGGAAGTCAGCAAAACACAGCAAGAGCTGTGCCCCACATCACACCTAATGAGTCCTAGGGGAAGGCTTATGTCCCATGAACTCCATAAAGTGTCTTGTGCCTTAAGGATTGATGAAGAAGATCTTTGACTTAAGCAAGACCCCCCACAGAAGGCTGTGGGCCCCCAGAGAGACCTTGGGAAAGTCTTAGACTCCAAAGAAATCCCAGGGAAGTCTTTAGTCACCAAAGAAACCCTGGAAAGGCCTTAGATCCCAGTAAGAACCTGGGAAGGTCTTTGGTCATATTGAAACCCAGAGAAGACCTTAATTTGTTACAGTAGGGAGGGCTTTTGTGCCACTGGGACCCTGTCAAGGTTTTAGTTTCCAGTGAGCTC

**Th-2A Cre #4 (+1 bp)**

ACGAAGTGGAAGCTAAGAGCTGCCATGAGGGCAAAGGTTATAGAAGGGGTAGGCAGTACAAATTCAAGGGACACAGTTATACAGGGTCTGAGAAAGGAAGAGGAGACTGGAAAGTTGGTCCTTATCACTTCTCCTGGCCTGCTGTCCTGGCATGTCCACTTCAGTTTAGGGACTCTCTAGGGCACACAGTCAAGGAGAGCCTCCTGAGGGAAGAAGTCCAGATGAGAAATGAAGTGCTTTGCAGAAGTCTGCCCACAAGATTTTCTGGGGCTTTAGTCTCCTGAATGTCAGGAATGGGCTTTAGGGGTTTGGCAAACTCAGAAAAGCCATTTCCCCATTCTCCTGGAGGTTGCAGATTTTATCCCGAGCTACCTGACTCTAGCCTGCTTTCTCACCCTTACATCCAGTCTCCTGTGATTGAGGAATCATTACCGGCTTCCCTCCACAGGAACTATGCCTCTCGTATCCAGCGCCCATTCTCTGTGAAGTTTGACCCGTACACACTGGCCATTGACGTACTGGACAGCCCTCACACCATCCAGCGCTCCTTGGAGGGGGTCCAGGATGAGCTGCACACCCTGGCCCACGCACTGAGTGCAATTAGCGGAAGCGGAGAGGGCAGAGGAAGTCTGCTAACATGCGGTGACGTGGAGGAGAATCCCGGCCCTATGCCCAAGAAGAAGAGGAAGGTGTCCAATTTACTGACCGTACACCAAAATTTGCCTGCATTACCGGTCGATGCAACGAGTGATGAGGTTCGCAAGAACCTGATGGACATGTTCAGGGATCGCCAGGCGTTTTCTGAGCATACCTGGAAAATGCTTCTGTCCGTTTGCCGGTCGTGGGCGGCATGGTGCAAGTTGAATAACCGGAAATGGTTTCCCGCAGAACCTGAAGATGTTCGCGATTATCTTCTATATCTTCAGGCGCGCGGTCTGGCAGTAAAAACTATCCAGCAACATTTGGGCCAGCTAAACATGCTTCATCGTCGGTCCGGGCTGCCACGACCAAGTGACAGCAATGCTGTTTCACTGGTTATGCGGCGGATCCGAAAAGAAAACGTTGATGCCGGTGAACGTGCAAAACAGGCTCTAGCGTTCGAACGCACTGATTTCGACCAGGTTCGTTCACTCATGGAAAATAGCGATCGCTGCCAGGATATACGTAATCTGGCATTTCTGGGGATTGCTTATAACACCCTGTTACGTATAGCCGAAATTGCCAGGATCAGGGTTAAAGATATCTCACGTACTGACGGTGGGAGAATGTTAATCCATATTGGCAGAACGAAAACGCTGGTTAGCACCGCAGGTGTAGAGAAGGCACTTAGCCTGGGGGTAACTAAACTGGTCGAGCGATGGATTTCCGTCTCTGGTGTAGCTGATGATCCGAATAACTACCTGTTTTGCCGGGTCAGAAAAAATGGTGTTGCCGCGCCATCTGCCACCAGCCAGCTATCAACTCGCGCCCTGGAAGGGATTTTTGAAGCAACTCATCGATTGATTTACGGCGCTAAGGATGACTCTGGTCAGAGATACCTGGCCTGGTCTGGACACAGTGCCCGTGTCGGAGCCGCGCGAGATATGGCCCGCGCTGGAGTTTCAATACCGGAGATCATGTGCCTCAGTAGAGCCAGGATCAAGCTGGTGGCTGGACCAATGTAAATATTGTCATGAACTATATCCGTAACCTGGATAGTGAAACAGGGGCAATGGTGCGCCTGCTGGAAGATGGCGACTAAACTAGTCTGTGCCTTCTAGTTGCCAGCCATCTGTTGTTTGCCCCTCCCCCGTGCCTTCCTTGACCCTGGAAGGTGCCACTCCCACTGTCCTTTCCTAATAAAATGAGGAAATTGCATCGCATTGTCTGAGTAGGTGTCATTCTATTCTGGGGGGTGGGGTGGGGCAGGACAGCAAGGGGGAGGATTGGGAAGACAATAGCAGGCATGCTGGGGATGCGGTGGGCTCTATGGGCGGCCGCTAGTGCATAGGGTACCACCCACAGGTGCCAGGGGCCTTTCCCAAAGTCTCCATCCCCTTCTCCAACCTTTCCTGGCCCAGAGGCTTTCCCATGTGTGTGGCTGGGCCCTTTGATGGGCTCCTCTTGGACCCCCATCCTCCCAACACTGCTTCTCAACCATGTCTTACTACTGCATGCACTCCAGGGTGGTCCTGCATTCCTCCTGCCCTCCATGCTCTATACTACCCTGATTATTCTCTCAATAAAGGAAGGAAAGATCTCCAGGGCTGCCTCTGGTCACTGCGTCACCACAAAGATAGTTCCTAGCATTCATATCTGCTCTGGCTTCACTTGGAGGTTTAGACGGTTTCTTCTCCCCCACCTCTTTCTTCCAATTCAGCCTCCTGGACACTTCAGACCAGACAGAAGGTACTGGTGTTATCTCCCTGCCAACTATGAGCTCTGAAACAGTACTGGGGGGTGTTGAACAAGGCCCCAGATGAGCTGCCCCAGAAGCATGGGCTAACAACACAAAGGTGGAGACCAGGGTAAGGAGTGACAGTCACAGCTGTGCTCACCAGGGAACAGCCTCTGACAAGGCTTGTAGACCAAGCCCTCTCCCTGTGCTCAGGAGTCTTAGGCACCTTGCAAGTAGGCAGAGGATTGAAGGAAGTCAGCAAAACACAGCAAGAGCTGTGCCCCACATCACACCTAATGAGTCCTAGGGGAAGGCTTATGTCCCATGAACTCCATAAAGTGTCTTGTGCCTTAAGGATTGATGAAGAAGATCTTTGACTTAAGCAAGACCCCCCACAGAAGGCTGTGGGCCCCCAGAGAGACCTTGGGAAAGTCTTAGACTCCAAAGAAATCCCAGGGAAGTCTTTAGTCACCAAAGAAACCCTGGAAAGGCCTTAGATCCCAGTAAGAACCTGGGAAGGTCTTTGGTCATATTGAAACCCAGAGAAGACCTTAATTTGTTACAGTAGGGAGGGCTTTTGTGCCACTGGGACCCTGTCAAGGTTTTAGTTTCCAGTGAGCTC

**Th-2A Cre #5 (+1 bp)**

ACGAAGTGGAAGCTAAGAGCTGCCATGAGGGCAAAGGTTATAGAAGGGGTAGGCAGTACAAATTCAAGGGACACAGTTATACAGGGTCTGAGAAAGGAAGAGGAGACTGGAAAGTTGGTCCTTATCACTTCTCCTGGCCTGCTGTCCTGGCATGTCCACTTCAGTTTAGGGACTCTCTAGGGCACACAGTCAAGGAGAGCCTCCTGAGGGAAGAAGTCCAGATGAGAAATGAAGTGCTTTGCAGAAGTCTGCCCACAAGATTTTCTGGGGCTTTAGTCTCCTGAATGTCAGGAATGGGCTTTAGGGGTTTGGCAAACTCAGAAAAGCCATTTCCCCATTCTCCTGGAGGTTGCAGATTTTATCCCGAGCTACCTGACTCTAGCCTGCTTTCTCACCCTTACATCCAGTCTCCTGTGATTGAGGAATCAATACCGGCTTCCCTCCACAGGAACTATGCCTCTCGTATCCAGCGCCCATTCTCTGTGAAGTTTGACCCGTACACACTGGCCATTGACGTACTGGACAGCCCTCACACCATCCAGCGCTCCTTGGAGGGGGTCCAGGATGAGCTGCACACCCTGGCCCACGCACTGAGTGCAATTAGCGGAAGCGGAGAGGGCAGAGGAAGTCTGCTAACATGCGGTGACGTGGAGGAGAATCCCGGCCCTATGCCCAAGAAGAAGAGGAAGGTGTCCAATTTACTGACCGTACACCAAAATTTGCCTGCATTACCGGTCGATGCAACGAGTGATGAGGTTCGCAAGAACCTGATGGACATGTTCAGGGATCGCCAGGCGTTTTCTGAGCATACCTGGAAAATGCTTCTGTCCGTTTGCCGGTCGTGGGCGGCATGGTGCAAGTTGAATAACCGGAAATGGTTTCCCGCAGAACCTGAAGATGTTCGCGATTATCTTCTATATCTTCAGGCGCGCGGTCTGGCAGTAAAAACTATCCAGCAACATTTGGGCCAGCTAAACATGCTTCATCGTCGGTCCGGGCTGCCACGACCAAGTGACAGCAATGCTGTTTCACTGGTTATGCGGCGGATCCGAAAAGAAAACGTTGATGCCGGTGAACGTGCAAAACAGGCTCTAGCGTTCGAACGCACTGATTTCGACCAGGTTCGTTCACTCATGGAAAATAGCGATCGCTGCCAGGATATACGTAATCTGGCATTTCTGGGGATTGCTTATAACACCCTGTTACGTATAGCCGAAATTGCCAGGATCAGGGTTAAAGATATCTCACGTACTGACGGTGGGAGAATGTTAATCCATATTGGCAGAACGAAAACGCTGGTTAGCACCGCAGGTGTAGAGAAGGCACTTAGCCTGGGGGTAACTAAACTGGTCGAGCGATGGATTTCCGTCTCTGGTGTAGCTGATGATCCGAATAACTACCTGTTTTGCCGGGTCAGAAAAAATGGTGTTGCCGCGCCATCTGCCACCAGCCAGCTATCAACTCGCGCCCTGGAAGGGATTTTTGAAGCAACTCATCGATTGATTTACGGCGCTAAGGATGACTCTGGTCAGAGATACCTGGCCTGGTCTGGACACAGTGCCCGTGTCGGAGCCGCGCGAGATATGGCCCGCGCTGGAGTTTCAATACCGGAGATCATGTGCCTCAGTAGAGCCAGGATCAAGCTGGTGGCTGGACCAATGTAAATATTGTCATGAACTATATCCGTAACCTGGATAGTGAAACAGGGGCAATGGTGCGCCTGCTGGAAGATGGCGACTAAACTAGTCTGTGCCTTCTAGTTGCCAGCCATCTGTTGTTTGCCCCTCCCCCGTGCCTTCCTTGACCCTGGAAGGTGCCACTCCCACTGTCCTTTCCTAATAAAATGAGGAAATTGCATCGCATTGTCTGAGTAGGTGTCATTCTATTCTGGGGGGTGGGGTGGGGCAGGACAGCAAGGGGGAGGATTGGGAAGACAATAGCAGGCATGCTGGGGATGCGGTGGGCTCTATGGGCGGCCGCTAGTGCATAGGGTACCACCCACAGGTGCCAGGGGCCTTTCCCAAAGTCTCCATCCCCTTCTCCAACCTTTCCTGGCCCAGAGGCTTTCCCATGTGTGTGGCTGGGCCCTTTGATGGGCTCCTCTTGGACCCCCATCCTCCCAACACTGCTTCTCAACCATGTCTTACTACTGCATGCACTCCAGGGTGGTCCTGCATTCCTCCTGCCCTCCATGCTCTATACTACCCTGATTATTCTCTCAATAAAGGAAGGAAAGATCTCCAGGGCTGCCTCTGGTCACTGCGTCACCACAAAGATAGTTCCTAGCATTCATATCTGCTCTGGCTTCACTTGGAGGTTTAGACGGTTTCTTCTCCCCCACCTCTTTCTTCCAATTCAGCCTCCTGGACACTTCAGACCAGACAGAAGGTACTGGTGTTATCTCCCTGCCAACTATGAGCTCTGAAACAGTACTGGGGGGTGTTGAACAAGGCCCCAGATGAGCTGCCCCAGAAGCATGGGCTAACAACACAAAGGTGGAGACCAGGGTAAGGAGTGACAGTCACAGCTGTGCTCACCAGGGAACAGCCTCTGACAAGGCTTGTAGACCAAGCCCTCTCCCTGTGCTCAGGAGTCTTAGGCACCTTGCAAGTAGGCAGAGGATTGAAGGAAGTCAGCAAAACACAGCAAGAGCTGTGCCCCACATCACACCTAATGAGTCCTAGGGGAAGGCTTATGTCCCATGAACTCCATAAAGTGTCTTGTGCCTTAAGGATTGATGAAGAAGATCTTTGACTTAAGCAAGACCCCCCACAGAAGGCTGTGGGCCCCCAGAGAGACCTTGGGAAAGTCTTAGACTCCAAAGAAATCCCAGGGAAGTCTTTAGTCACCAAAGAAACCCTGGAAAGGCCTTAGATCCCAGTAAGAACCTGGGAAGGTCTTTGGTCATATTGAAACCCAGAGAAGACCTTAATTTGTTACAGTAGGGAGGGCTTTTGTGCCACTGGGACCCTGTCAAGGTTTTAGTTTCCAGTGAGCTC

**Supplementary Figure 2. Sequenceing data of funder mice and rats.**

| **Supplementary Table 1. Target sequences for sgRNAs** | | |
| --- | --- | --- |
| *Gene* |  | Sequence (5' to 3') |
| *Kcnab1* | sgRNA-1 | AAAGGACTATAGATCATA |
|  | sgRNA-2 | GTATAAATGACTGCTTAATG |
| *Mc4r* | sgRNA-1 | GTTGTCTAGCAGGTATTAAG |
|  | sgRNA-2 | GTGGAACCTTGATAAATAAC |
| *Ctgf* | sgRNA-1 | CGGAGACATGGCGTAAAGCC |
|  | sgRNA-2 | GACATAGGGCTAGTCTACAA |
| *Slc12a1* | sgRNA-1 | CACAACATTTCTCATCCTTC |
|  | sgRNA-2 | AACTTAAGGAGAGCCCGCTG |
| *Bmi1* | sgRNA-1 | TGAATGAAGGTAACTAAATC |
|  | sgRNA-2 | CAATCTGTATGCCTAAAAAG |
| *Plxnd1* | sgRNA-1 | CCTGGTCACTCTTCTGCATC |
|  | sgRNA-2 | GGGGTGTTGGCACTTTGATG |
| *Cdkn2a* | sgRNA-1 | CCCAAGAGCAGAGCTAAATC |
|  | sgRNA-2 | AAGCACAAGCGGCTATCGCG |
| *Pva* | sgRNA-1 | TTGGCGGGCCAGAACCTCAG |
|  | sgRNA-2 | TCTGGTGGCCGAAAGCTAAG |
| *Th* | sgRNA-1 | CTGTGATTGAGGAATCATAC |
|  | sgRNA-2 | GTGCCATTAGCTAAATGCAT |
